# An Early Enriched Experience Drives an Activated Microglial Profile at Site of Corrective Neuroplasticity in Ten-m3 Knock-Out Mice

**DOI:** 10.1523/ENEURO.0162-22.2022

**Published:** 2023-01-03

**Authors:** Lara Rogerson-Wood, Justin Petersen, Abe Kairouz, Claire S. Goldsbury, Atomu Sawatari, Catherine A. Leamey

**Affiliations:** School of Medical Sciences (Neuroscience), FMH, University of Sydney, New South Wales 2006, Australia

**Keywords:** binocular, enrichment, microglia, plasticity, repair, Teneurin 3 (Ten-m3/Odz3)

## Abstract

Environmental enrichment (EE) is beneficial for brain development and function, but our understanding of its capacity to drive circuit repair, the underlying mechanisms, and how this might vary with age remains limited. Ten-m3 knock-out (KO) mice exhibit a dramatic and stereotyped mistargeting of ipsilateral retinal inputs to the thalamus, resulting in visual deficits. We have recently shown a previously unexpected capacity for EE during early postnatal life (from birth for six weeks) to drive the partial elimination of miswired axonal projections, along with a recovery of visually mediated behavior, but the timeline of this repair was unclear. Here, we reveal that with just 3.5 weeks of EE from birth, Ten-m3 KOs exhibit a partial behavioral rescue, accompanied by pruning of the most profoundly miswired retinogeniculate terminals. Analysis suggests that the pruning is underway at this time point, providing an ideal opportunity to probe potential mechanisms. With the shorter EE-period, we found a localized increase in microglial density and activation profile within the identified geniculate region where corrective pruning was observed. No comparable response to EE was found in age-matched wild-type (WT) mice. These findings identify microglia as a potential mechanistic link through which EE drives the elimination of miswired neural circuits during early postnatal development. Activity driven, atypical recruitment of microglia to prune aberrant connectivity and restore function may have important therapeutic implications for neurodevelopmental disorders such as autistic spectrum disorder.

## Significance Statement

This study identifies microglia as having potential mechanistic involvement in the removal of early miswired neural projections and associated improvement in function. Importantly, we find that this reparative process is driven by enhanced levels of experience during an early window of postnatal life. Given the evidence for etiological involvement of axonal guidance and pathfinding errors in neurodevelopmental disorders, these results may have important therapeutic implications for these conditions.

## Introduction

Environmental enrichment (EE) presents the potential to induce many beneficial effects in the nervous system. It has been shown to delay the onset of neurodegenerative disease (Hannan, 2014; Gelfo et al., 2018; Wassouf and Schulze-Hentrich, 2019), promote recovery following nervous system injury (McDonald et al., 2018), and ameliorate the impact of multiple neurodevelopmental disorders (Consorti et al., 2019). Despite the wealth of evidence for EE’s positive neurologic influence, knowledge of its underlying mechanisms remains limited.

Work performed using the rodent visual pathway has shown that EE accelerates neural development (Cancedda, 2004; Ciucci et al., 2007; BS Wang et al., 2013) and promotes plasticity across the lifespan, enabling recovery from detrimental sensory manipulations during early life (Sale et al., 2007; Tognini et al., 2012; Baroncelli et al., 2013; Levine et al., 2017). The capacity for EE to correct neural wiring deficits caused by gene mutations affecting critical proteins, such as those involved in axonal guidance and synaptogenesis, has not been widely considered though. This avenue of research is important, as genetically driven neural miswiring is thought to underlie many neurodevelopmental disorders (Jain et al., 2020; Kennedy et al., 2020). We have previously shown that long-term EE induces pruning which partially repairs (Eggins et al., 2019) the aberrant wiring caused by genetic knock-out (KO) of the axonal guidance/synaptogenesis associated transmembrane protein Ten-m3 (Leamey et al., 2007). This presents an excellent opportunity to probe candidate mechanisms by which EE might rescue genetically impaired neural circuitry and function.

Ten-m3 has a major role in establishing the innervation pattern of ipsilateral retinal inputs to the dorsal lateral geniculate nucleus (dLGN; for review, see Leamey and Sawatari, 2019). In Ten-m3 KO mice, the initial ingrowth of ipsilateral retinal inputs to the dLGN, occurring around birth [from, embryonic day (E)18 to postnatal day (P)3; Godement et al., 1984], is markedly aberrant (Glendining et al., 2017), despite the segregation of ipsilateral and contralateral retinal inputs occurring within the normal time course (Glendining et al., 2017). Consequently, ipsilateral retinal inputs to the dLGN terminate in a ventrolaterally elongated strip (Leamey et al., 2007), rather than in a discrete, dorsomedial (DM) patch seen in wild-type (WT) mice (Godement et al., 1980; Jaubert-Miazza et al., 2005). This altered mapping disrupts the visual topography usually present in the dLGN (Reese and Jeffery, 1983; Piscopo et al., 2013) and downstream primary visual cortex (Merlin et al., 2013). Visually-mediated behavior is also severely impaired in Ten-m3 KO mice (Leamey et al., 2007; Blok et al., 2020). The beneficial effect of long-term EE on these aberrant phenotypes was recently demonstrated. When applied for six weeks from birth, EE elicited significant removal of the most visuotopically-mismapped ipsilateral retinal inputs to the dLGN, but was noted not to influence the initial ingrowth of axons (Eggins et al., 2019). Improvements in visually-mediated behavior were also seen (Blok et al., 2020). The cellular mechanisms which might facilitate this corrective pruning remain unknown.

Microglia, the brain’s main immune cell population, are also key mediators of structural neuroplasticity (Ferro et al., 2021). Microglia have been shown to prune retinogeniculate inputs during normal development, facilitating the early segregation of ipsilateral and contralateral terminals between P5 and P9 (Schafer et al., 2012), as well as a later axonal refinement at ∼P40–P50 (Schafer et al., 2016). EE has been shown to modulate microglial function, altering gene and/or protein expression, microglial morphology, and phagocytic behavior in response to multiple forms of immune insult (Xu et al., 2016; Garofalo et al., 2017; Hase et al., 2017; Ziegler‐Waldkirch et al., 2018; Cope et al., 2019). Little has been shown though of EE’s effects on microglial-mediated plasticity. We hypothesised that microglia might be involved in mediating the EE-driven corrective pruning of ectopic RGCs previously seen in six-week-old Ten-m3 KO mice.

To address this possibility, we first sought a more accurate timeline for the EE-induced pruning of miswired projections in Ten-m3 KO mice, and found that it had commenced and was in process, 3.5 weeks after birth. A significant improvement in visually-mediated-behavior of enriched KOs was also observed at this age. Most notably, the anatomical pruning correlated both temporally and spatially with a localized increase in microglia density and activation profile in enriched Ten-m3 KO mice. These results provide the first evidence of microglia as having potential mechanistic involvement in an EE-driven repair of miswired neural circuitry and restoration of neural function.

## Materials and Methods

All procedures were performed with approval from the University of Sydney Animal Ethics Committee and conformed with the National Health and Medical Research Council (Australia) guidelines for the care and use of laboratory animals. All mice were housed in climate-controlled rooms (∼23.50°C, 40–70% humidity) at a University of Sydney Laboratory Animal Services (LAS) facility on a fixed 12/12 h light/dark cycle. Standard mouse chow and water were provided *ad libitum*.

### Mice

Original generation of the Ten-m3 KO line has been previously described (Leamey et al., 2007). Experimental homozygous [Ten-m3 −/− (KO)] mice were obtained through the crossing of heterozygous females with heterozygous or homozygous males. Breeding was undertaken in standard environment (SE) housing with SE parentage. A few days before birth, pregnant dams were transferred from their breeding cages into EE or, maintained in SE (for detailed housing descriptions, see Eggins et al., 2019; Blok et al., 2020). Powdered standard mouse chow mixed with drinking water and “Necta H_2_O” hydration gel (Able Scientific) were included in the cage *ad libitum* from postnatal day (P)14 to provide support near weaning. Dams were removed from litter cages when pups were approximately P21. Litters were of mixed genotypes: heterozygotes, homozygotes, and wild-types. To distinguish, genotyping was undertaken (between P10 and P21) using tail and/or ear biopsies as previously described (Leamey et al., 2007). Mice of both sexes were included in the study in approximately equal numbers, according to availability.

### Behavioral testing

SE (*n* = 8 KO and *n* = 8 WT) and EE (*n* = 8 KO and *n* = 8 WT) mice were tested for visually-mediated behavior using the looming stimulus. The procedure used was based on that described previously (Yilmaz and Meister, 2013; Blok et al., 2020). Briefly, an aquarium (48 × 48 × 30 cm) with a shelter (12.5 × 10.5 × 7.5 cm) placed in one corner was used as the test chamber. All walls and the floor were covered in red-colored Perspex. An LCD panel (27-inch diagonal; LG 27MP37HQ) was placed on top of the chamber to present the stimulus. Because of the size difference between the monitor and aquarium, the portion of the top of the chamber not covered by the panel effectively defined a dark region within the arena. This dark region was contiguous with the placement of the shelter. A Petri dish (6 cm diameter) containing a sunflower seed was positioned in the center of the chamber (directly below stimulus center). This was used to entice the mice to the center of the box, thus allowing the stimulus to be presented consistently within the binocular visual field. All experiments were run under the illumination of a red light (72 W, 240 V).

The looming stimulus was generated using PyschoPy2. The stimulus consisted of a black circle on a gray background, which expanded from 2° to 20° of visual angle over a period of 250 ms (expansion rate of 72°/s). The expanded disk remained on screen for an additional 250 ms, after which it was cleared leaving just the gray background. The stimulus was then repeated 15 times, with a 500-ms interstimulus interval between each cycle. The stimulus was triggered manually when the mouse’s nose breached the edge of the Petri dish in the middle of the testing chamber.

The mice were placed individually inside the test chamber for 10 min to habituate on the day before testing. Conditions were identical to the experimental paradigm apart from the absence of the visual stimulus (i.e., with only the gray background projected onto the screen). Mice were then returned to their home-cage and the test chamber was cleaned thoroughly with 70% ethanol.

On the following day, mice were placed inside the test chamber and allowed to habituate for 2 min. Following this period, the stimulus was triggered as soon as the mouse’s nose breached the edge of the Petri dish. All behavior experiments were performed between 10 A.M. and 12 P.M. to minimize variation due to Circadian rhythms.

### Video analysis

The videos were coded, and subsequent analysis was performed by an investigator blinded to genotype and housing condition. They were sampled at 6 Hz using a custom script generated in Python (Python Software Foundation) and analyzed using the plugin MtrackJ module of ImageJ (NIH). At the beginning of each video, the *x* and *y* coordinates of the perimeter of the testing chamber, center, and shelter were defined and recorded. The tracking began 2 s before stimulus onset, and ended the frame just after the stimulus terminated, or when the mouse escaped, whichever came first. The tip of the mouse’s snout was used to track the position of the mouse in each frame. An escape was considered to have occurred when the mouse’s nose proceeded past the coordinates which defined the entrance to the shelter. This meant that if a mouse entered the dark area next to the shelter, this was also considered an escape (Blok et al., 2020).

The following parameters were extracted: latency (the time taken) from stimulus onset to escape, distance traveled from stimulus onset to escape, prestimulus mean velocity (over the 20 s before the stimulus onset), poststimulus mean velocity from stimulus onset to escape, and latency (time taken) from stimulus onset to commencement of escape (determined manually by changes in direction and velocity poststimulus onset). Subjects that did not escape had their latency capped at 11.5 s. An additional parameter which assessed the efficiency of the escape trajectory was calculated from the ratio of the actual distance traveled to the straight-line distance between the starting (the position of the subject at stimulus onset) and stopping points (position at escape or end of the assessment period). This gave an optimal value of 1 for the most efficient trajectories possible (less efficient trajectories yielded higher values).

### Anterograde tracing and immunofluorescence labeling

On P25–P26, experimental mice used for anatomical tracing received intraocular injections of the anterograde tracer Cholera Toxin Subunit B (Recombinant), Alexa Fluor 488 and 594 conjugate (Invitrogen, catalog #C22841 or #C22842, 1% w/v in 0.1 m PB) into the posterior chamber of each eye as previously described (Eggins et al., 2019). Twenty-four to 48 h later, mice were euthanased with an overdose of sodium pentobarbitol (>100 mg/kg, i.p.), transcardially perfused (Eggins et al., 2019), postfixed, and their brains sectioned coronally (60-μm thickness) on a freezing microtome. The entire rostrocaudal extent of the dLGN was mounted and analyzed. For microglial analysis, only the left eye was injected at P24, and mice were euthanized 24 h later. Six sections from each mouse, which encompassed the rostral third of the dLGN ipsilateral to the injected eye (the region where the ventrolateral mis-mapping of ipsilateral retinal terminations is most prominent; Leamey et al., 2007) were chosen for subsequent processing. Sections were rinsed (0.1 m PB with 0.3% Triton X-100; PB-Tx), incubated for 2 h in blocking solution (2% v/v NGS in PB-Tx, at room temperature) and then placed in primary antibody incubation solution (rabbit anti-Iba-1, Wako, catalog #1919741, 1:500 and rat anti-CD68, Abcam, catalog #ab53444, 1:1000 in blocking solution) overnight (18–26 h at room temperature). Following another rinse (PB-Tx) and incubation in secondary antibody solution for 2 h (goat anti-rabbit Alexa Fluor 594, Invitrogen, catalog #A11037 or goat anti-rabbit Alexa Fluor 594, Invitrogen, catalog #R37117 or goat anti-rabbit Alexa Fluor 488, Invitrogen, catalog #A11008 and goat anti-rat Alexa Fluor 647, Invitrogen catalog #A21247, 1:500 in blocking solution, room temperature), sections were rinsed a final time (PB-Tx), mounted and cover-slipped using Prolong Diamond mountant (Invitrogen, catalog #P36965). After curing (∼24 h), coverslip edges were sealed with clear nail varnish and the slides stored chilled (<5°C) until imaging.

### Microscopy

For anatomical studies, the entire series of dLGN sections was photographed using a Zeiss Deconvolution microscope as previously described (Eggins et al., 2019). For the microglial studies, two of the most rostral dLGN sections, excluding the dLGN section abutting the optic tract, were chosen from each mouse for imaging. Low-power confocal z-stacks [5 μm z-stack interval for KO cohort, *n* (EE-KO) = 2, *n* (SE-KO) = 3; 1.2 μm z-stack interval for WT cohort, *n* (EE-WT) = 4, *n* (SE-WT) = 4] were first acquired for qualitative data (WT cohort was also used for fold-change analysis). This was undertaken on a Zeiss LSM 800 confocal microscope using a 20× NA0.8 Plan-Apochromat dry objective. Acquisition of high-power confocal z-stacks for single microglia analysis [0.4 μm z-stack interval for KO cohort; 0.5 μm z-stack interval for WT cohort; *n* (EE-KO) = 5, *n* (SE-KO) = 5; *n* (EE-WT) = 4, *n* (SE-WT) = 4] was then performed at the ventrolateral border of the CTB-labeled RGC ipsilateral terminations in the dLGN [defined as the region of interest (ROI); visible via anterograde tracing]. Most samples [*n* (EE-KO) = 2, *n* (SE-KO) = 3; *n* (EE-WT) = 4, *n* (SE-WT) = 4] were acquired on a Zeiss LSM 800 confocal microscope using a 63× NA1.40 Plan-APOchromat oil immersion objective (laser excitation line 488 nm for Alexa Fluor 488, 561 nm for Alexa Fluor 594, and 640 nm for Alexa Fluor 647). Others were captured on a Leica SPEII confocal microscope [*n* (EE-KO) = 3; *n* (SE-KO) = 1] using a 63× NA1.30 ASC APO oil immersion objective (laser excitation line 488 nm for Alexa Fluor 488, 532 nm for Alexa Fluor 594, and 635 nm for Alexa Fluor 647), or a Zeiss LSM 510 Meta confocal microscope [*n* (SE-KO) = 1] using a 63× NA1.40 Plan-APOchromat oil immersion objective (laser excitation line 488 nm for Alexa Fluor 488, 561 nm for Alexa Fluor 594, and 633 nm for Alexa Fluor 647). Use of the range indicator function on each scope helped ensure images acquired on different microscopes had similar intensity levels. An additional set of low-power confocal z-stacks of a second KO cohort [2.32-μm z-stack interval*; n* (EE-KO) = 5, *n* (SE-KO) = 5] were used for the fold-change analysis. This imaging was undertaken on a Zeiss LSM 800 confocal microscope using a 10× NA0.45 Plan-APOchromat dry objective.

### Image analysis

Image analysis was undertaken blind to housing condition. For anatomical tracing studies, this was performed as described previously (Eggins et al., 2019). Three sections from the rostral dLGN were analyzed from each mouse. Total dLGN area and the ipsilateral terminal area were measured using ImageJ (NIH). For the regional analysis, the dLGN was rotated such that the long dorsomedial-ventrolateral axis of the dLGN was horizontal and the nucleus was divided into thirds (Dorsal (D), Middle (M), and Ventral (V) regions) along this axis. For microglial studies (fold change and single-cell Imaris measures), analysis was done on two rostral dLGN sections from each mouse.

Fold change analysis was undertaken using ImageJ software (FIJI, NIH). Low-power confocal z-stacks first underwent a background fluorescence subtraction using the rolling-ball background correction function. A rolling ball radius of 15 for the Ionized-calcium binding adaptor-moleule-1 (Iba1) channel and 10 for the CD68 channel was used for images acquired on the 20× objective, and a radius of 10 for Iba-1 channel and 5 for the CD68 channel for images acquired on the 10× objective. Maximum intensity projections (MIPs) were then acquired for the Iba-1, CD68, and CTB (labeled retinal terminals) channels and saved as a composite image. From these, ROIs (80 × 80 μm around the ventrolateral or dorsomedial border of CTB-labeled ipsilateral retinal terminals) and dLGN outlines were defined and saved for each section by using the ROI manager tool. Binary images were created for the CD68 and Iba-1 channels for each section through using the inbuilt “default” (CD68 channels) or “moments” (Iba-1 channels) thresholding algorithm function in ImageJ. For each binary image (CD68 and Iba-1 channels), the measurements of “average particle size” and “%area” were taken for the whole dLGN and its associated ROI, and a fold change ratio calculated (ROI/whole dLGN).

For the Imaris-based single-cell microglial analysis, high-power confocal z-stacks were first preprocessed using ImageJ (FIJI, NIH) software. An attenuation correction plugin (Biot et al., 2008) was first applied to compensate for the decrease in fluorescence intensity with increased imaging depth (all channels). Background fluorescence was then subtracted (all channels) using the rolling-ball background correction function. A rolling ball radius of 50 for the Iba-1 channel and of 15 for the CTB and CD68 channels was used. Some z-stacks also had the “De-speckle” and/or “Remove Outliers” function applied (choice guided by the type of background noise present). A previous study suggested that applying a mean filter assists with the surface rendering of microglia in Imaris (Schafer et al., 2012, 2014). As such, a mean filter (size of 1.5) was additionally applied to Iba-1 channels. Preprocessed images were transferred from FIJI to Imaris (Oxford Instruments) for quantitative analysis using the *Image from FIJI* function of the ImarisXT Imaris- ImageJ/FIJI bridge.

To determine microglial number within each ROI (for microglial density measurements) and identify relevant microglia for subsequent quantitative analysis, a manual count of individual microglia (indicated by Iba-1 expression, to visualise morphology) was undertaken using the three-dimensional (3D) visualization and manipulation capacities of Imaris (surpass mode). Only microglia which had a whole cell body contained entirely within the ROI, and not abutting the border, were included in the count and subsequent quantitative analysis.

To facilitate the automatic calculation of specific immunofluorescence volumes (described below), 3D surface rendering was undertaken for 3D solidity, arborized volume, %CD68, and average lysosome volume measures. This was achieved using the automatic surface creation tool within Imaris. Absolute intensity thresholding was used with the “smooth” function enabled. Absolute intensity thresholding values for each fluorescence channel from each section were determined empirically from an initially established reference example (to maintain rendering consistency). To further avoid biasing the output values, this process was undertaken before commencement of experimental surface rendering.

3D solidity was a measure of microglial morphology designed specifically for this study, to better integrate the other 3D measurements. The method was modelled off a previously published two-dimensional version which was found to successfully and quantitatively distinguish between more ramified (lower 3D solidity) versus more amoeboid (higher 3D solidity) microglial morphologies (Xu et al., 2016, 2018). For this measure, the semi-automatic Autopath Mode of Imaris Filament Tracer was first used to outline the filament structure of each microglia. For each microglial filament, a 3D convex hull (minimum bounding three-dimensional convex polygon) was automatically generated using the convex hull Xtension package (available from Imaris Open). To isolate each microglia for analysis, Iba-1 fluorescence outside of its associated convex hull was masked (to exclude from subsequent analysis). The remaining Iba-1 fluorescence was surface rendered (surface area detail 0.210 μm), the irrelevant surfaces (those not part of current microglia undergoing analysis) manually deleted, and the Imaris “unify surfaces” function applied (to facilitate automatic recognition of multiple disjointed surfaces as part of one volume). 3D solidity measures were derived from the resultant volumetric measurements (microglia Iba-1 volume/convex hull volume; calculated per microglia).

Arborized volume was included as another measure of microglial morphology, as it is intrinsically less sensitive to methodological variability compared to surface rendering or Iba-1 labeling. This measure aimed to give a quantitative assessment of the expanse of microglial arborization by measuring the volume of each microglia’s associated convex hull (see above), with larger hull volumes being indicative of microglial arborization taking up a greater expanse of brain tissue. Arborized volume was determined for each microglia.

For the %CD68 measure, the approach was modelled off a previously published protocol (Schafer et al., 2012, 2014). The measure was aimed at assessing the overall CD68 expression in microglia, normalized to microglial volume. Each microglial Iba-1 surface rendering had its external CD68 fluorescence masked. The remaining CD68 fluorescence was surface rendered (surface area detail 0.1 μm) and the “unify surfaces” function applied. %CD68 measures were derived from the resultant volumetric measurements (CD68 volume/microglia Iba-1 volume × 100%; calculated per microglia).

Average lysosome volume measure was included to quantitatively elaborate on the %CD68 results. It aimed to quantitatively elucidate whether the average size of the lysosomes (indicated by CD68 expression) was altered between the difference cohorts of mice. For this measure, the CD68 surface rendering for each microglia was “split” (to facilitate automatic recognition of disjointed CD68 volumes and lysosomes, as individual volumes). Average lysosome volume was derived from the resultant volumetric measures (individual CD68 volumes below 0.1 μm^3^, largely background fluorescence, were excluded from all averages).

### Experimental design and statistical analysis

For all analyses, mice used came from a minimum of three different litters. Sample sizes were chosen based on previous studies. Pups of both sexes were included in the analysis in approximately equal numbers. All analyses were performed blind as to treatment group.

Microsoft excel, SPSS and MATLAB were used for data processing and graphical representations (significance level α set to 0.05). For behavioral studies, each mouse is represented by a single data point. For anatomical tracing, three sections from the rostral part of the dLGN were used in each case. Each data point represents a value from a single section. A mixed model analysis was used to adjust for the use of multiple sections from each mouse. For microglial studies two sections from rostral dLGN were used for each subject. Each mouse was represented by a single data point for cells/mm^3^ measurements. These values were subject to standard pairwise comparisons (see Results). All microglia (variable numbers) whose cell body was contained entirely within the ROI (116 × 116 microns around the ventrolateral retinogeniculate terminal border) and was not directly abutting the ROI border was included in the analysis. The remaining microglial measures (%CD68, arborized volume, 3D solidity, and average lysosome volume) involved individual cell or section (fold change) analysis and as such, multiple cell or section replicates were used. For ease of statistical analysis, aggregate values were acquired for each mouse. Means were used for %CD68, 3D solidity, and fold change measures. Median aggregate values were used for the average lysosome volume measure because of the obvious presence of outliers (single microglia) in some mouse cell replicate datasets. These summary measures were then subject to standard univariate omnibus testing (see Results). Both raw and summary values for microglial studies are depicted on relevant figures.

## Results

### A 3.5-week exposure to EE from birth promotes rescue of a Ten-m3 KO-induced visually mediated behavioral response defect

**Figure 1.**
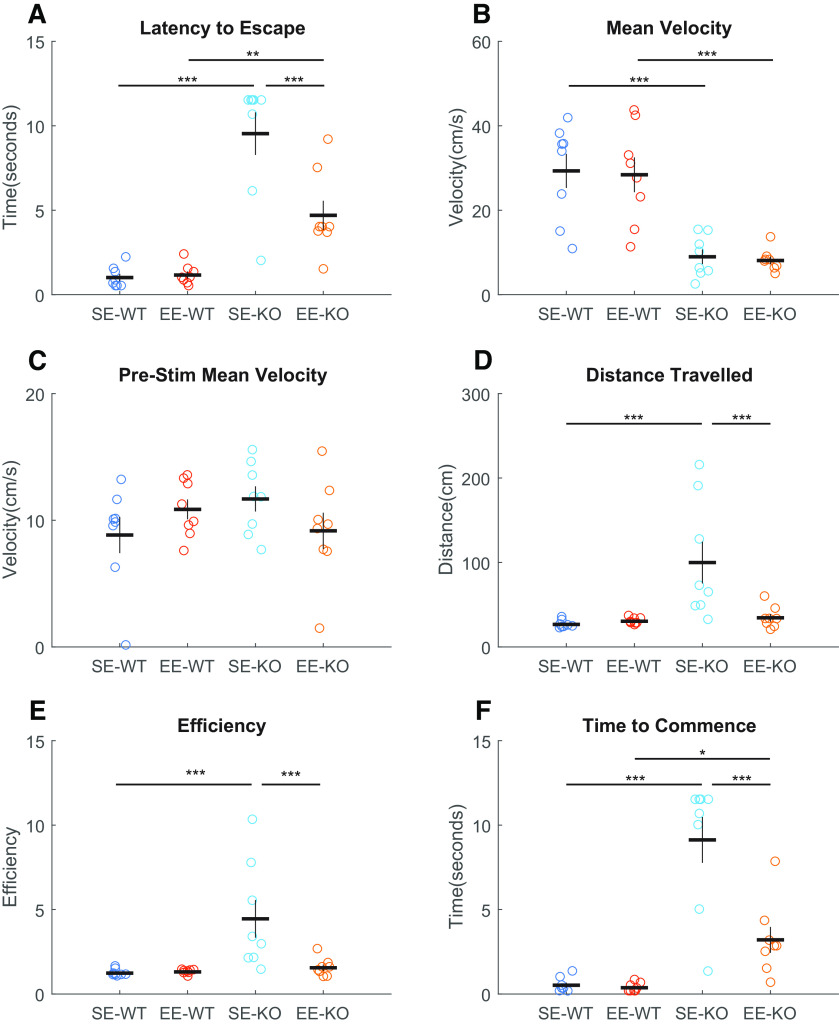
Approximately three weeks of EE from birth induces partial recovery of visually mediated flight behavior in Ten-m3 KOs. Latency from initiation of the looming stimulus to escape (***A***) is significantly longer in KOs compared with WTs for both SE (*p *<* *0.001) and EE (*p *=* *0.003) conditions. EE KOs, however, exhibited a significantly reduced time to escape compared with SE KOs (*p *<* *0.001). Mean velocity (***B***) is significantly greater in WTs compared with KOs for both SE (*p *<* *0.001) and EE (*p *<* *0.001) cohorts. No differences between housing groups within genotypes were detected. Mean velocity before stimulus initiation (***C***) did not differ significantly for any groups. Distance traveled (***D***) is significantly different between SE groups (WTs < KOs; *p *<* *0.001) but not between EE-WTs and EE-KOs. Consistent with this, EE-KOs traveled detectably shorter distances compared with SE-KOs (*p *=* *0.001). ***E***, The efficiency of the escape trajectory (actual distance traveled/straight line distance between start and finish points; optimum value of 1 with high values indicating lower efficiency) was significantly less for SE-KOs compared to SE-WTs (*p* < 0.001). EE-KOs, however, were more efficient than SE-KOs (*p* = 0.001). ***F***, Time to commence flight is significantly higher in KOs compared with WTs, both in SE (*p *<* *0.001) and EE (*p *=* *0.016) conditions. This value is significantly decreased in EE compared SE KOs (*p *<* *0.001). Results shown are of pairwise comparisons (Bonferroni corrected for multiple comparisons). Each dot signifies a single animal, eight animals per group. Mean is indicated by the horizontal line and the SEM is shown by the vertical lines. ***p ≤* *0.001, **p ≤* *0.01, *p ≤ 0.05.

Ten-m3 KO mice exhibit highly stereotyped miswiring of ipsilateral retinogeniculate axons with associated profound deficits in binocularly mediated visual behavior (Leamey et al., 2007; Blok et al., 2020). We first asked whether exposure to EE from birth was sufficient to drive recovery of the response to a visually-mediated behavioral task in Ten-m3 KOs by P25–P26. Following presentation of the looming stimulus (a rapidly expanding dark disk presented to the dorsal visual field emulating the approach of an aerial predator (Blok et al., 2020), marked differences in responses were observed between Ten-m3 KOs compared with WT controls, as well as between standard-housing (SE) and EE raised KOs (Fig. 1). All SE-WT mice reacted robustly, fleeing rapidly from the center of the chamber (mean latency of escape was 1 s) with no impact of EE on this cohort (Fig. 1*A*).

SE-KO mice did not respond reliably, with many continuing to explore the chamber for several seconds following stimulus presentation. Accordingly, their mean escape latency was substantially longer, >9 s. EE from birth (until P25/26) partially rescued this behavior, with EE-KOs showing a mean latency to escape of approximately half that of age-matched SE-KOs, although they did not reach WT performance levels. Statistical testing (univariate ANOVA with genotype and housing as fixed factors) confirmed significant effects of genotype (*F*_(1,28)_ = 59.94, *p *<* *0.001), housing (*F*_(1,28)_ = 9.066, *p *=* *0.005), and genotype × housing interaction (*F*_(1,28)_ = 10.244, *p *=* *0.003). Pairwise comparisons revealed that SE-KOs (9.535 ± 0.779, *n* = 8) took significantly longer (*p *<* *0.001) to escape than SE-WTs (1.015 ± 0.779 s; mean ± SEM, *n* = 8). Similarly, EE-KOs (4.699 ± 0.779, *n* = 8) also took significantly longer (*p *=* *0.003) than EE WTs (1.163 ± 0.779, *n* = 8) to reach the shelter. No differences (*p *=* *0.894) were detected between SE-WTs and EE-WTs. EE-KOs, however, took significantly less time to escape compared with SE-KOs (*p *<* *0.001; Fig. 1*A*).

Given the known deficits exhibited by the Ten-m3 KO mice (Leamey et al., 2007; Tran et al., 2015); the EE-dependent changes in escape latency could arise because of a number of factors including improvements in sensory perception as well as mobility. In order to see which of these may be primarily responsible for the rescue of visually-mediated behavior, we examined the degree to which potentially relevant parameters were affected by EE. This analysis indicated that changes in motor ability were unlikely to be responsible, as while mean poststimulus velocity was significantly reduced in Ten-m3 KOs compared with WTs, there was no impact of EE (Fig. 1*B*). Omnibus testing confirmed an effect of genotype (*F*_(1,28)_ = 44.570, *p *<* *0.001). Pairwise comparisons revealed that SE-WTs (mean ± SEM, 29.310 ± 3.044, *n* = 8) exhibited significantly greater velocities compared with SE-KOs (8.971 ± 3.044, *n* = 8; *p *<* *0.001). These values were not impacted by EE (EE-WT: 28.398 ± 3.044, *n* = 8; EE-KO: 8.098 ± 3.044, *n* = 8; *p *<* *0.001). No within-genotype differences were detected across housing (SE-WT vs EE-WT: *p *=* *0.834; SE-KO vs EE-KO: *p *=* *0.841).

Motor deficits or lack of exploration of the chamber were also unlikely to be involved, as the mean velocity of ambulation before stimulus initiation exhibited no differences across genotype or housing (Fig. 1*C*; genotype: *F*_(1,28)_ = 0.236, *p *=* *0.631; housing: *F*_(1,28)_ = 0.043, *p *=* *0.837; genotype × housing interaction: *F*_(1,28)_ = 3.628, *p *=* *0.067; pairwise comparisons between genotypes within housing groups: *p *>* *0.100; pairwise comparisons between housing groups within genotypes: *p *>* *0.140). Therefore, the differences between both the genotypes and housing groups emerge only after stimulus presentation.

Distance traveled following stimulus presentation (Fig. 1*D*) showed an effect of genotype, but unlike velocity, this was impacted by housing. No apparent differences in distance traveled were observed between SE-WTs (26.623 ± 12.582, *n* = 8) and EE-WTs (30.444 ± 12.582, *n* = 8). EE-KOs (34.509 ± 12.582, *n* = 8), however, EE-KOs traveled notably shorter distances compared with SE-KOs (99.931 ± 12.582, *n* = 8). Interestingly, EE completely abrogated the difference between KOs and WTs when distance was considered. Statistical testing for distance confirmed significant effects of genotype (*F*_(1,28)_ = 9.454, *p *=* *0.005), housing (*F*_(1,28)_ = 5.993, *p *=* *0.021), and a genotype × housing interaction (*F*_(1,28)_ = 7.572, *p *=* *0.010). Pairwise comparisons confirmed that SE-KOs traveled significantly greater distances (*p *<* *0.001) compared with SE-WTs. No differences (*p *=* *0.832) were observed between SE-WTs and EE-WTs. EE-KOs, however, traveled significantly shorter distances compared with SE-KOs (*p *=* *0.001), suggesting an EE-induced difference in this group. EE eliminated the difference between KOs and WTs with respect to distance (*p *=* *0.821).

Path efficiency was also assessed and gave essentially the same results as distance traveled (Fig. 1*E*). No differences were noted between SE and EE-WTs, but EE-KOs exhibited markedly more efficient trajectories compared with SE-KOs. For efficiency, omnibus testing also showed significant effects of genotype (*F*_(1,28)_ = 9.280, *p *=* *0.005), housing (*F*_(1,28)_ = 6.221, *p *=* *0.019), and a genotype × housing interaction (*F*_(1,28)_ = 6.840, *p *=* *0.014). SE-KOs (4.452 + 0.567, *n* = 8) were significantly less efficient (*p *<* *0.001) in seeking shelter compared with SE-WTs (1.240 ± 0.567, *n* = 8). No differences (*p *=* *0.763) were observed between EE-KOs (1.553 ± 0.567, *n* = 8) and EE-WTs (1.309 ± 0.567, *n* = 8). No differences (0.932) were detected between SE-WTs and EE-WTs. EE-KOs, however, exhibited significantly more efficient trajectories compared with SE-KOs (*p *=* *0.001). These findings indicate that housing had little impact on the overall mobility of Ten-m3 KOs, but rather influenced their trajectory following stimulus presentation.

We also examined the latency to commence flight (from stimulus initiation) as a proxy for stimulus processing time (Fig. 1*F*). For WTs, the mean time to commence escape was a fraction of a second (SE-WTs: 0.520 ± 0.785 s, *n* = 8; EE-WTs: 0.375 ± 0.785 s, *n* = 8). In contrast, many SE-KOs did not initiate any obvious escape response to this stimulus within the available time (9.124 ± 0.785 s, *n* = 8). Consistent with an effect of enrichment, EE-KOs exhibited a reduced time to commence escape (3.206 ± 0.785 s, *n* = 8) compared with SE-KOs, suggesting that they took less time to process the stimulus and/or initiate an escape response (although they did not reach WT levels of performance). These results suggest that the behavior changes due to EE was primarily affecting sensory processing/response planning rather than motor function in Ten-m3 KO mice. Omnibus testing (univariate ANOVA with genotype and housing as fixed factors) revealed significant effects of genotype (*F*_(1,28)_ = 53.073, *p *<* *0.001), housing (*F*_(1,28)_ = 14.918, *p *= 0.001), and a genotype × housing interaction (*F*_(1,28)_ = 13.525, *p *=* *0.001). Pairwise comparisons indicated that SE-KOs took significantly more time (*p *<* *0.001) before flight commencement compared with SE-WTs. Similarly, EE-KOs took more time (*p *=* *0.016) compared with EE-WTs. No differences (*p *=* *0.897) were detected between SE and EE WTs. EE-KOs, however, required significantly less time before flight commenced compared with SE-KOs (*p *<* *0.001).

Together, these findings show that even at just 3.5 weeks of age, WT mice exhibit robust responses to a looming stimulus presented overhead. Further, EE from birth to this age point was sufficient to drive a partial, but significant, rescue of visually-mediated flight behavior in Ten-m3 KOs. This EE-induced rescue was driven by a reduced processing time and selection of more efficient escape trajectories. Since the stimulus can be perceived only visually, these changes are consistent with a partial recovery of functional vision.

### Exposure to EE for 3.5 weeks from birth is sufficient to induce removal of miswired retinal terminals in Ten-m3 KO mice

**Figure 2.**
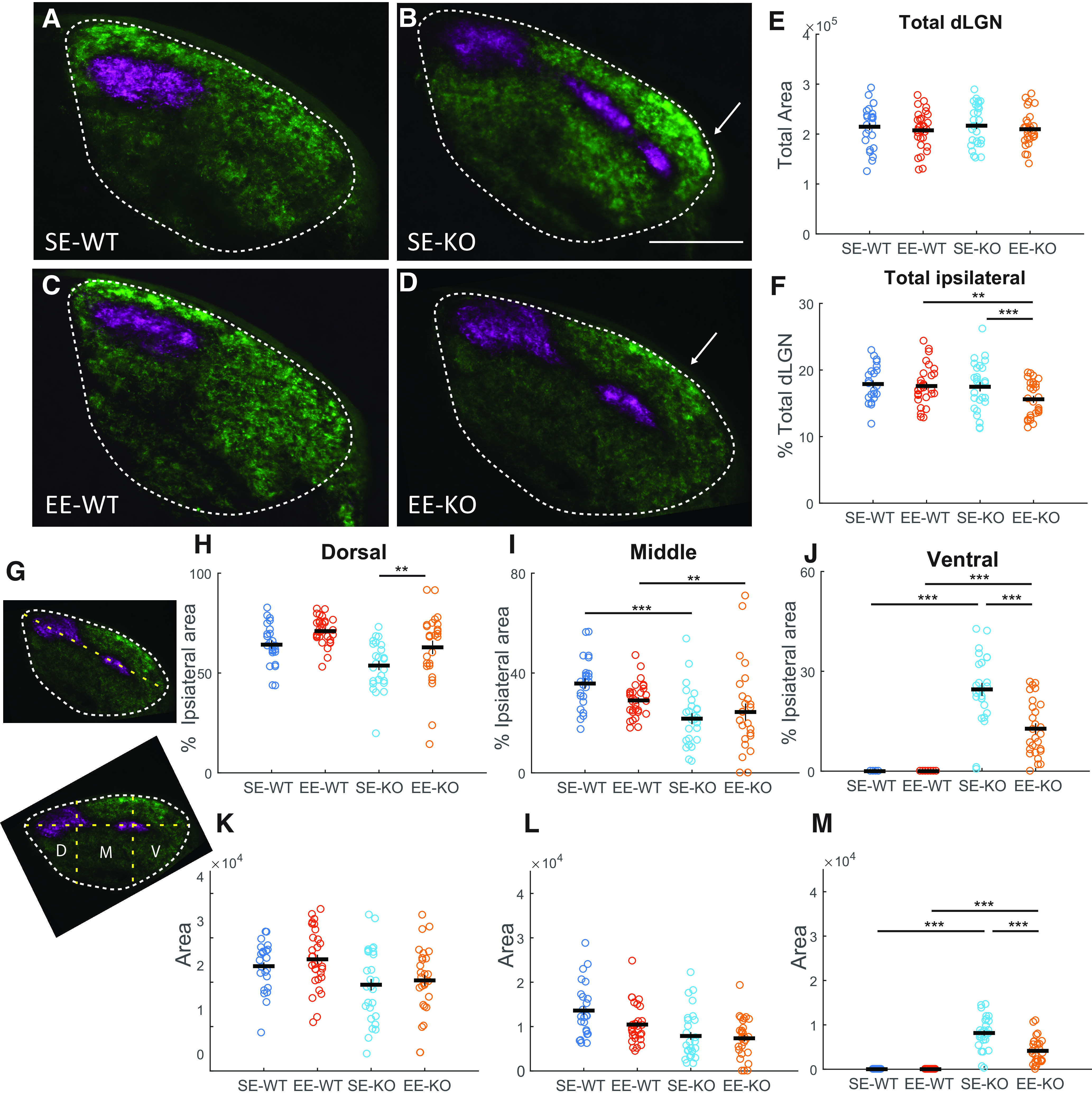
EE for 3.5 weeks from birth is able to drive pruning of miswired ipsilateral projections. ***A–D***, Coronal sections through the dLGN at P26 following anterograde transport of CTB from the retina. Contralateral terminals are shown in green and ipsilateral in magenta. In SE-WTs (***A***), ipsilateral terminals are confined to dorsolateral dLGN (dotted line), whereas in SE-KOs (***B***), they are distributed in a narrow band which approaches the ventrolateral border of the dLGN. Arrow indicates ventrolateral extremity of ipsilateral projection. No change is apparent following EE in WTs (***C***). In EE-KOs (***D***), the ipsilateral projection does not extend as far ventrolaterally as SE-KOs (arrow). ***E***, Analysis of the total area of the dLGN showed no differences with respect to housing or genotype. ***F***, There was a slight but significant reduction in the percentage of the dLGN occupied by ipslateral terminals in EE-KOs compared with EE-WTs (*p *=* *0.005) and SE-KOs (*p *<* *0.001). ***G***, Shows the long dorsomedial-ventrolateral axis of the dLGN (upper) and how this was divided arithmetically into dorsal (D) middle (M), and ventral (V) thirds along this axis to determine the locus of changes. ***H***, While no differences in the proportion of ipsilateral terminals targeting dorsal dLGN was detected between genotypes. EE induced a significant increase in this value for KOs (*p *=* *0.002). ***I***, While WTs exhibited a significantly greater proportion of ipsilateral terminals in the middle third compared to KOs for both SE (*p *=* *0.001) and EE (*p *=* *0.006) conditions, enrichment did not induce any changes within genotypes. ***J***, KOs exhibited increased proportions of ipsilateral terminals in the ventral third of the dLGN compared with WTs for both SE (*p *<* *0.001) and EE (*p *<* *0.001) conditions. EE significantly reduced this value within KOs (*p *<* *0.001). The absolute area occupied by ipsilateral terminals in dorsal (***K***), middle (***L***), and ventral (***M***) thirds of the dLGN was also analyzed. This showed a significantly more terminals in ventral dLGN in SE-KOs (*p* < 0.001) and EE-KOs (*p* = 0.001) compared with respective WTs. Importantly, there was a significant decrease in the area of the ipsilateral terminals targeting ventral dLGN in EE-KOs compared with SE-KOs (*p* < 0.001). In ***E***, ***F***, ***H–M***, each dot represents a single section, three sections were analyzed per dLGN, five to seven animals per condition. Horizontal and vertical lines mark mean and SEM respectively. The *y*-axis on ***E*** and ***K–M*** is shown in arbitrary units squared. Results shown are of pairwise comparisons (Bonferroni corrected for multiple comparisons). **p ≤* *0.01, ***p ≤* *0.001. Orientation: dorsal to the top and lateral to the right. Scale bar: 200 μm (applies to ***A–D***).

We next asked whether the rescue of visually-mediated behavior we observed was accompanied by an anatomical correction of miswired ipsilateral terminals in the dLGN. While this was previously observed following six weeks of EE from birth (Eggins et al., 2019; Blok et al., 2020), whether a shorter period of EE led to similar changes was unknown. Anterograde tracing revealed the expected elongation of ipsilateral retinal axons into the far ventrolateral dLGN in SE-KOs (*n* = 5; Fig. 2*B*, arrow), which differed markedly from SE-WTs (*n* = 5; Fig. 2*A*), where these terminals were confined to the dorsomedial part of the dLGN (see also Leamey et al., 2007). As noted previously, the miswiring of ipsilateral projections was most marked in the rostral third of the dLGN and thus our analysis focused on this region. Following 3.5 weeks of EE (from birth) there was a notable retraction of ipsilateral terminals from the ventrolateral part of the dLGN in Ten-m3 KOs (*n* = 5; Fig. 2*D*, arrow). There was no obvious impact of EE on WT retinal axonal projections to the dLGN (*n* = 7; Fig. 2*C*). Quantification confirmed these observations. There was no effect of either genotype or housing on total dLGN area (Fig. 2*E*; *F*_(1,154.095)_ = 0.130, *p* = 0.719; housing: *F*_(1,154.095)_ = 0.271, *p* = 0.603; interaction: *F*_(1,154.095)_ = 0.001, *p* = 0.973). When considering total ipsilateral terminal areas as a percentage of the dLGN area, however, omnibus testing revealed that there was a significant effect of housing (*F*_(1,15.511)_ = 15.387, *p *=* *0.001; Fig. 2*F*). An interactive effect of genotype and housing was also detected (*F*_(1,15.511)_ = 6.246, *p *=* *0.024). Pairwise comparisons showed that ipsilateral terminals took up significantly less area (*p *= 0.005) in EE-KOs (15.59 ± 0.52%) compared with EE-WTs (17.59 + 0.53%), but there was no difference between SE-WTs (17.88 + 0.55%) and SE-KOs (17.20 ± 0.84%). When compared between housing conditions, EE-KOs had significant less ipsilateral area compared with SE-KOs (*p *< 0.001), but EE-WTs and SE-WTs did not differ from each other. These findings demonstrate that EE from birth can mediate a significant enhancement in the pruning of aberrant ipsilateral projections in KOs by just 3.5 weeks of age.

To confirm the locus of the EE-driven reduction in ipsilateral input, the proportion of ipsilateral label contained within the dorsal (D), middle (M), or ventral (V) regions of the dLGN was determined by dividing the long (dorsomedial-ventrolateral axis of the dLGN) into thirds (see Materials and Methods; Fig. 2*G*; Eggins et al., 2019). The most dorsal region exhibited an effect of housing as a percentage of the total ipsilateral label (*F*_(1,20.358)_ = 15.10, *p *=* *0.001; Fig. 2*H*). Pairwise comparisons showed that enriched values (69.75 ± 2.27%) were significantly greater (*p *=* *0.002) than those of standard housed subjects (58.30 ± 2.27%), but only for KOs. For the middle region, an effect of genotype, but not of housing was observed (*F*_(1,20.261)_ = 24.108, *p *< 0.001; Fig. 2*I*). Pairwise comparisons revealed that for both standard (*p *=* *0.001) and enriched conditions (*p *=* *0.006), KO values (SE: 21.58 ± 2.15%; EE: 19.635 ± 2.15%) were significantly less than WTs (SE: 33.82 ± 2.35%; EE: 28.41 ± 1.90%). Finally, for the ventral region, effects for genotype (*F*_(1,15.933)_ = 370.575, *p *<* *0.001), housing (*F*_(1,15.933)_ = 39.60, *p* < 0.001), and genotype × housing interaction (*F*_(1,15.933)_ = 39.604, *p *<* *0.001; Fig. 2*J*) were detected for the percentage of ipsilateral label. Pairwise comparisons revealed that for both standard (*p *<* *0.001) and enriched conditions (*p *< 0.001), KO values (SE: 18.82 ± 0.723%; EE: 9.55 ± 0.72%) were significantly greater than WTs (SE: 0.00 ± 0.76%; EE: 0.00 ± 0.74%). Comparing within genotypes, only KOs showed detectable differences, with EE-KOs exhibiting significantly smaller values compared with SE-KO mice (*p *<* *0.001).

This analysis showed an EE-induced increase in the proportion of ipsilateral label in dorsal and a decrease in ventral KO dLGN. In order to further investigate the basis for this, the ipsilateral areas were analyzed in absolute terms, to prevent the potential confound of a significant EE-induced decrease in total ipsilateral label influencing the result. We found that there was no change in the area of the dorsal region occupied by ipsilateral terminals with respect to either housing or genotype (Fig. 2*K*; genotype: *F*_(1,4.94)_ = 6.312, *p* = 0.054; housing: *F*_(1,4.94)_ = 0.681, *p* = 0.447; interaction: *F*_(1,4.94)_ = 0.001, *p* = 0.980). Similarly, there was no change in the middle region (Fig. 2*L*; genotype: *F*_(1,0.245)_ = 8.494, *p* = 0.557; housing: *F*_(1,0.245)_ = 0.890, *p* = 0.723; interaction: *F*_(1,0.245)_ = 0.390, *p* = 0.786). In the ventral region however, significant effects of genotype, housing, and a genotype × housing interaction were observed (Fig. 2*M*; genotype: *F*_(1,8.054)_ = 138.469, *p* < 0.001; housing: *F*_(1,8.054)_ = 14.883, *p* = 0.005; interaction: *F*_(1,8.054)_ = 14.883, *p* = 0.005). Pairwise differences were also found with WT values significantly lower than KO for both SE (*p* < 0.001) and EE conditions (*p* = 0.001). Comparing within genotypes, only KOs showed differences with EE-KOs having significantly lower values than SE-KOs (*p* < 0.001).

Thus, the only part of the dLGN to exhibit an EE-driven change in ipsilateral terminal area in both relative and absolute terms was the ventrolateral region of Ten-m3 KO mice, where a significant reduction was detected. This confirms the ventrolateral region as the primary locus of observed EE-enhanced pruning. The EE-induced increase in the proportion, but not the absolute amount, of ipsilateral label in the dorsal dLGN is most likely a secondary effect of the decline of label in ventral dLGN. Importantly, the reduced labeling in the ventral region of EE-KOs was consistent and highly significant in both analyses, confirming this as a region of primary interest.

Further, we noticed that the appearance of the labeled ipsilateral terminals at the ventrolateral extremity appeared qualitatively different in EE-KOs with a narrow, trailing band of labeled projections (Fig. 2*D*, arrow) rather than the wider, more abrupt ending which was typical of SE-KOs (Fig. 2*B*, arrow). The narrow, trailing band at the far ventrolateral border of the ipsilateral projection in EE-KOs suggested that pruning may be actively underway in this region. This was not seen previously following six weeks of EE (Eggins et al., 2019). Further, while the loss of ipsilateral terminals from ventrolateral dLGN was significant in EE-KOs compared with SE-KOs, the magnitude of the change appeared qualitatively less than what we had observed following six weeks of EE (Eggins et al., 2019).

**Figure 3.**
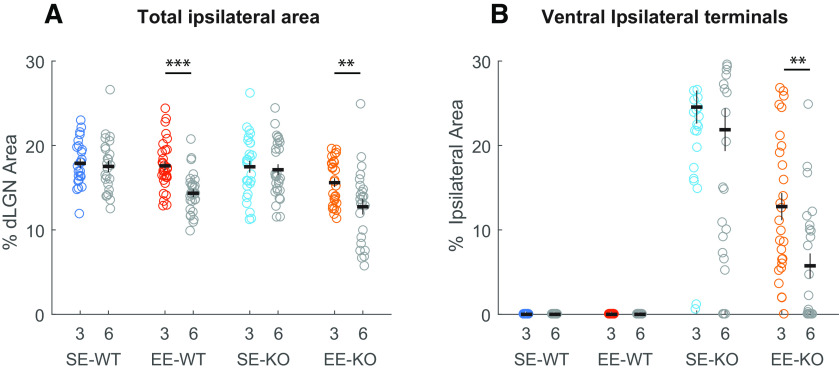
The EE-induced pruning of mismapped ipsilateral retinogeniculate projections is more extensive at 6 than 3.5 weeks. Graphs compare data from 3.5-week age point (colored circles as in Fig. 2; marked as three weeks on *x*-axis because of space restrictions) to that previously obtained at six weeks (gray; taken from Eggins et al., 2019). ***A***, Ipsilateral terminal area occupies a significantly smaller percentage of the dLGN in 6-week compared with 3.5-week EE-KOs (*p* = 0.004) There is also a reduction in 6-week EE-WTs compared with 3.5-week EE-WTs (*p* = 0.001). ***B***, The proportion of the ipsilateral terminal label targeting ventrolateral dLGN is significantly less in 6-week compared with 3.5-week EE-KOs (*p* = 0.010). No age-related changes are seen in other groups. Only age-related changes are shown here, other comparisons are shown in Figure 2 and as described by Eggins et al. (2019). ***p ≤* *0.001, **p ≤* *0.01, *p ≤ 0.05.

To address this possibility, we performed a statistical analysis comparing the data obtained here to key parameters from our previous data set, where a similar analysis was done following six weeks of EE from birth (Eggins et al., 2019). These data demonstrated that six-week-old EE mice (WTs and KOs) had significantly smaller percentage of the dLGN occupied by ipsilateral projection areas in the dLGN compared with 3.5-week-old EE mice (Fig. 3*A*; only age-related changes are shown). Omnibus testing for total ipsilateral terminal area (mixed model) revealed effects for housing (*F*_(1,52.957)_ = 43.897, *p *<* *0.001), age (*F*_(1,52.957)_ = 13.727, *p *=* *0.001), as well as interactions between genotype and housing (*F*_(1,52.957)_ = 5.231, *p *=* *0.026), and housing with age (*F*_(1,52.957)_ = 7.767, *p *=* *0.007). Pairwise comparisons revealed that six-week-old EE-WTs (14.35 ± 0.58%) had a slightly but significantly smaller percentage of the dLGN occupied by ipsilateral terminals compared with 3.5-week-old EE-WTs (*p *=* *0.001). Similarly, six-week-old EE-KOs (12.74 ± 0.92%) had significantly smaller total ipsilateral area compared with 3.5-week-old EE KOs (*p *=* *0.004).

Further, analysis confirmed an additional significant decrease in the proportion of ipsilateral label targeting the ventrolateral third of the dLGN following EE at 6 weeks compared with 3.5 weeks in KOs (Fig. 3*B*; only age-related changes are described here; for other comparisons see above and Eggins et al., 2019). Significant effects for genotype (*F*_(1,24.511)_ = 409.471, *p *<* *0.001), housing (*F*_(1,24.511)_ = 59.681, *p *<* *0.001) as well as an interaction between genotype and housing (*F*_(1,24.511)_ = 59.681, *p *<* *0.001) were found. Pairwise comparisons revealed that six-week EE-KOs had a significantly lower percentage of ipsilateral inputs targeting ventrolateral dLGN compared with 3.5-week-old EE-KOs (*p *=* *0.010). Together, these results strongly suggest that EE-induced pruning of miswired projections is underway at the ventrolateral extent of the ipsilateral terminals in the dLGN of Ten-m3 KOs at 3.5 weeks postnatal, providing both a spatial and temporal locus to probe the mechanism.

### Exposure to EE from birth for 3.5 weeks drives focalized microglial activation at the site of greatest ectopic RGC terminal removal in Ten-m3 KO mice

Given their previously characterized role as key mediators of the developmental pruning of WT RGC projections to the dLGN at other developmental ages (∼P4–P9; ∼P40–P50; Schafer et al., 2012, 2016) and demonstrated functional responsivity to EE in immune contexts (Xu et al., 2016; Garofalo et al., 2017; Hase et al., 2017; Ziegler‐Waldkirch et al., 2018; Cope et al., 2019), we asked whether microglia might be involved in the removal of miswired retinal projections occurring in EE-KOs (see also Eggins et al., 2019). To assess this possibility, an initial qualitative analysis was undertaken in SE-KOs (*n* = 5), EE-KOs (*n* = 5), SE-WTs (*n* = 4) and EE-WTs (*n* = 4) at ∼3.5 weeks of age (P25).

Ionized-calcium-binding-adaptor-molecule-1 (Iba-1), a cytoskeletal protein (Sasaki et al., 2001) predominantly expressed by microglial cells in the brain (Imai et al., 1996; Ito et al., 1998), and CD68, a lysosomal transmembrane glycoprotein present in microglia (Chistiakov et al., 2017) were used to visualize microglia. Because of its relatively even distribution throughout microglial cell bodies and processes (Sasaki et al., 2001), Iba-1 enables an effective assessment of overall microglial morphology. Morphology has been previously shown to be dramatically altered from the usual ramified appearance (small soma, extensive fine branches) in surveillant or resting microglia, to a more “amoeboid” (large soma, shorter fatter branches) appearance in microglia mediating elevated levels of structural pruning. CD68 has also been shown to dramatically increase and qualitatively change its expression pattern from small and punctate lysosomes, to large and “globular” in this same functional context (see Schafer et al., 2012).

**Figure 4.**
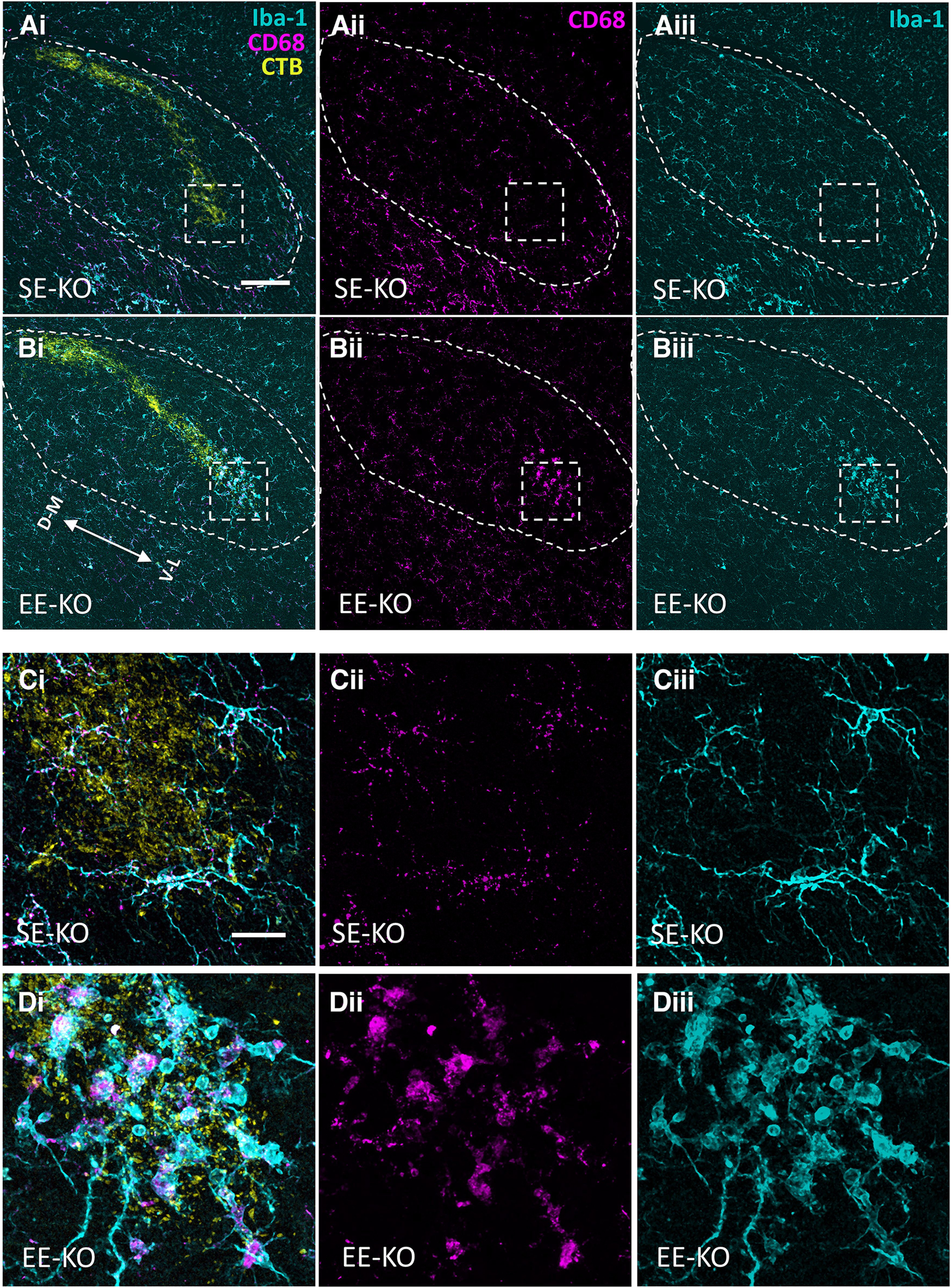
Environmental enrichment drives a visually apparent focal change in microglial Iba-1 and CD68 expression at the ventrolateral border of ipsilateral retinal terminals in the dLGN of Ten-m3 knock-out mice. ***A–D***, Low-power immunofluorescence images showing Iba-1 (cyan; microglial cytoskeletal marker) and CD68 (lysosomal marker, magenta) in coronal dLGN sections of a representative 3.5-week-old standard housed (SE-KO) and environmentally enriched (EE-KO) Ten-m3 knock-out mouse. Ipsilateral retinal terminals, labeled from the retina using an Alexa Fluor*-*conjugated CTB, are shown in yellow. Images are maximum intensity projections of confocal z-stacks. ***A***, Low-power images of a representative section from a SE-KO display a largely uniform distribution of Iba-1 or CD68 expression across the nucleus (see text). ***B***, Low-power images of a representative EE-KO display a distinct upregulation in the expression pattern of CD68 and Iba-1 in a small region of the nucleus: the ventrolateral (V–L) extremity of the ipsilateral RGC terminals (boxed). This focal change was seen clearly in all (5 out of 5) of the EE-KOs examined. ***C***, ***D***, Higher power images of this region of interest (ROI; boxed 116 × 116 μm) show microglia to have a higher density, altered morphology and increased CD68 expression in the representative EE-KO (***D***) compared with the SE-KO (***C***). Ventrolateral (V–L); dorsomedial (D–M). Scale bar: 100 μm (***A***, ***B***); 20 μm (***C***, ***D***).

Given the significant EE-driven anatomical pruning observed in Ten-m3 KOs at 3.5 weeks (see above), this cohort was qualitatively examined and compared with SE-KOs first. Iba-1 and CD68 immunofluorescence labeling, in combination with anterograde tracing, revealed SE-KOs as having largely uniform expression across the nucleus (Fig. 4*Ai–Aiii*). A very small amount of Iba-1 upregulation was observed in the ventrolateral dLGN in a single SE-KO examined (one of five), but no upregulation of CD68 was apparent (data not shown). When EE-KOs (Fig. 4*Bi–Biii*) were examined, however, a distinct and robust change in expression pattern of both markers (Iba-1 and CD68) was reliably observed (five of five cases) in a defined and consistent locus, the far ventrolateral extremity of ipsilateral RGC terminals (Fig. 4*Bi–Biii*, boxed). Both markers in this region (Fig. 4*Bi–Biii*, boxed), appeared to be markedly upregulated in their expression levels compared with all SE-KOs. Most importantly, this region of interest (ROI; Fig. 4, boxed region) was also the locus containing the most visuotopically mismapped projections, and coincides with the previously characterized site of anatomic repair in the ventrolateral dLGN (see above and Eggins et al., 2019).

Higher powered images revealed this change in the expression pattern of Iba-1 and CD68 observed around the ventrolateral border of retinal projections to the dLGN to be due to a collection of cellular changes. EE-KOs (Fig. 4*Di*,*Diii*) appeared to have an elevated microglial density compared with SE-KOs (Fig. 4*Ci*,*Ciii*). Cell morphologies were also phenotypically distinct in EE-KOs (Fig. 4*Di*,*Diii*) compared with those in SE-KOs. Microglia (as seen with Iba-1 labeling) in EE-KOs were much more irregular in their shape, not having the typical ramified appearance of those microglia typically seen in SE-KOs (Fig. 4*Ci*,*Ciii*). CD68 expression in EE-KOs was also generally increased, with the CD68 “clumps” larger and more “globular” (Fig. 4*Dii*) as opposed to the small and punctate CD68 clumps seen in most SE-KOs (Fig. 4*Cii*).

**Figure 5.**
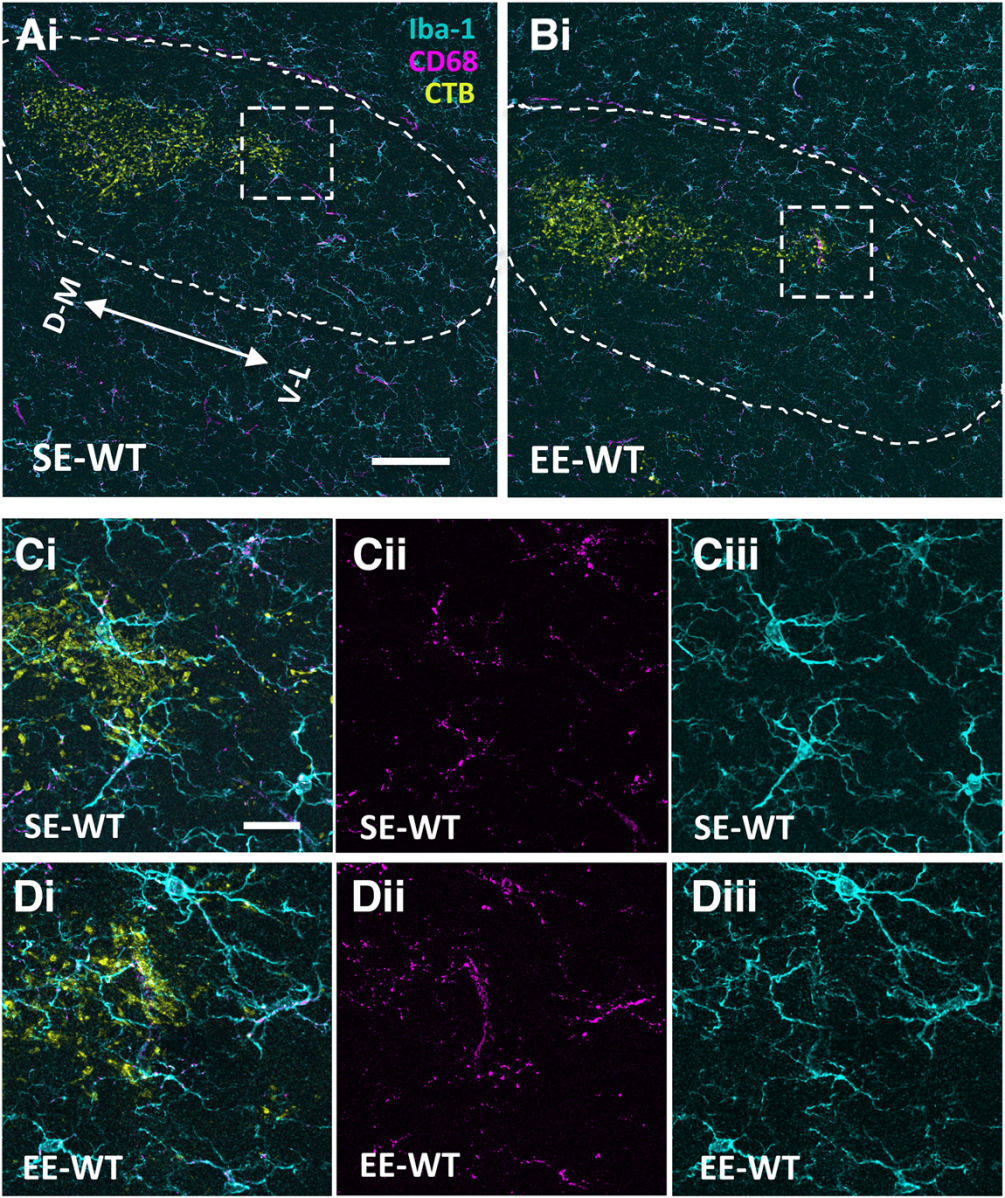
Environmental enrichment did not elicit any visually apparent changes to microglial Iba-1 or CD68 in the dLGN of wild-type mice. ***A–D***, Immunofluorescence images showing Iba-1 (cyan; microglial marker) and CD68 (magenta, lysosomal marker) in coronal dLGN sections of 3.5-week-old standard housed (SE-WTs) and environmentally enriched (EE-WT) wild-type mice. Ipsilateral retinal terminals, labeled from the retina using an Alexa Fluor-conjugated CTB, are shown in yellow. Images are maximum intensity projections of confocal z-stacks. ***A***, ***B***, Low-power images of a sample SE-WT and EE-WT show a largely uniform distribution of Iba-1 or CD68 expression across the nucleus, this was the case across all SE-WT (*n *=* *4) and EE-WTs (*n *=* *4) examined. ***C***, ***D***, High-power images of a region of interest (ROI) at the ventrolateral border of ipsilateral retinal projections (boxed in ***A*** and ***B***) show microglia as largely ramified in both SE-WTs and EE-WTs with no discernible differences in CD68 expression induced by EE. Ventrolateral (V–L); dorsomedial (D–M). Scale bar: 100 μm (***A***, ***B***); 20 μm (***C***, ***D***).

Age-matched EE-WT and SE-WT mice were examined and compared as a control since no significant EE-driven pruning of ipsilateral terminals was noted in this cohort at 3.5 weeks of age (see above). Iba-1 and CD68 immunofluorescence labeling, in combination with anterograde tracing (to definitively label ipsilateral RGC inputs) revealed a largely uniform expression of Iba-1 and CD68 expression across the nucleus (Fig. 5*A*,*B*) in all SE-WTs (*n* =* *4) and EE-WTs (*n* =* *4) examined. Higher-powered images (Fig. 5*C*,*D*) of the ventrolateral extremity of ipsilateral RGC projections (Fig. 5*A*,*B*) revealed microglia as being largely ramified in morphology (Fig. 5 *Ci*,*Ciii*,*Di*,*Diii*) with small punctate CD68 expression (Fig. 5*Cii*,*Dii*) in SE-WTs (Fig. 5*C*) and EE-WTs (Fig. 5*D*). No visually apparent differences were noted between the two housing conditions (SE and EE), consistent with the anatomical results for this cohort (see previous Results section). It should be noted that despite being more dorsomedially placed, this region is still the most anatomically equivalent region to the KO ROI, occurring at the ventral junction of ipsilateral and contralateral projections (and is henceforth referred as the WT ROI).

**Figure 6.**
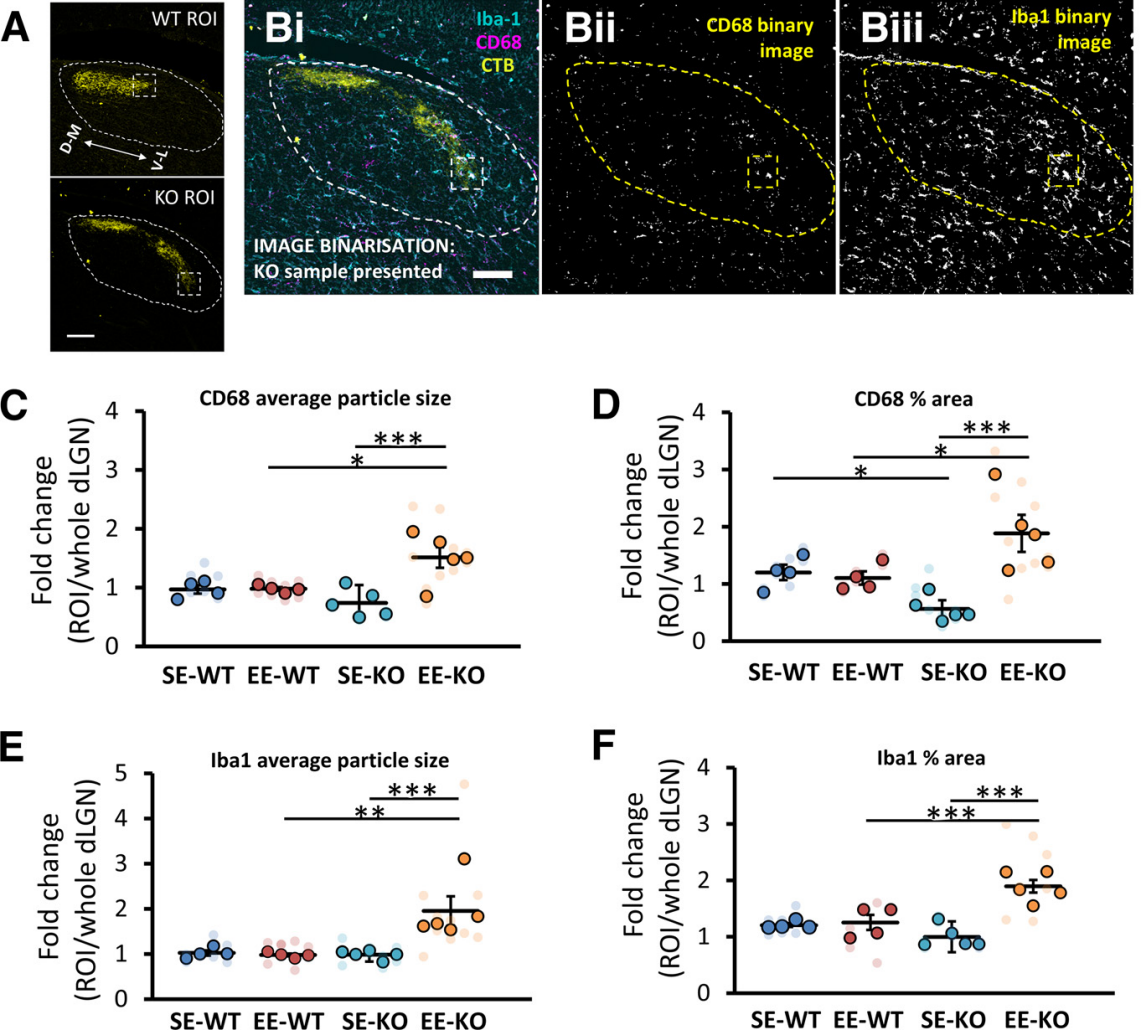
Environmental enrichment drives a significant increase in fold change expression of microglial CD68 and Iba-1 at the ventrolateral border of ipsilateral retinal terminals in Ten-m3 knock-out, but not wild-type mouse dLGNs. ***A***, Region of interest (ROI) in WT and KO mice (boxed region; 80 × 80 μm), the ventrolateral border of ipsilateral retinal terminals (yellow) in the dLGN (outlined). ***B***, Depiction of the image binarization process of the CD68 channel (***Bii***) and Iba-1 channel (***Biii***) image undertaken in ImageJ (see Materials and Methods). Sample image (***Bi***) shows immunofluorescence labeling of Iba-1 (cyan; microglial marker) and CD68 (magenta, lysosomal marker) in a coronal dLGN sections from a 3.5-week-old Ten-m3 KO mouse. Ipsilateral retinal terminals, labeled from the retina using an Alexa Fluor-conjugated CTB, are shown in yellow. ***C***, Fold change for “CD68 average particle size” was significantly greater in EE-KOs than SE-KOs (*p *=* *0.001) but was not significantly different between EE-WTs and SE-WTs (*p *=* *0.868). It was also significantly greater in EE-KOs than EE-WTs (*p *=* *0.021), but not significantly different between SE-KOs and SE-WTs (*p *=* *0.168). ***D***, Fold change for “CD68% area” was significantly greater in EE-KOs than SE-KOs (*p *<* *0.001) but was not significantly different between EE-WTs and SE-WTs (*p *=* *0.748). It was also significantly greater in EE-KOs than EE-WTs (*p *=* *0.013) and significantly lower in SE-KOs than SE-WTs (*p *=* *0.036). ***E***, Fold change for “Iba-1 average particle size” was significantly greater in EE-KOs than SE-KOs (*p *=* *0.001) but not significantly different between EE-WTs and SE-WTs (*p *=* *0.954). It was also significantly greater in EE-KOs than EE-WTs (*p *=* *0.002), but not significantly different between SE-KOs and SE-WTs (*p *=* *0.883). ***F***, Fold change for “Iba-1%” area was significantly greater in EE-KOs than SE-KOs (*p *<* *0.001) but not significantly different between EE-WTs and SE-WTs (*p *=* *0.771). It was also significantly greater in EE-KOs than EE-WTs (*p *=* *0.001), but not significantly different between SE-KOs and SE-WTs (*p *=* *0.167). Results shown are of pairwise comparisons. Intensely colored circles represent data values from independent samples: an aggregate value (see Materials and Methods). Faint data points, grouped in columns according to sample identity, represent data values from single section measurements. Four animals per housing condition for WTs and five animals per housing condition for KOs. Mean bars are depicted. Error bars show SEM. Ventrolateral (V–L); dorsomedial (D–M). Scale bars: 100 μm (***A***, ***Bi–Biii***). *p ≤* *0.05, **p ≤* *0.01, ***p ≤* *0.001.

To better capture the regionally focalised EE-driven upregulation in Iba-1 and CD68 expression pattern at the ventrolateral border of ipsilateral retinal terminals (ROI) in Ten-m3 KO mice (Fig. 4), a fold-change analysis of this ROI [*n* (EE-KO) = 5, *n* (SE-KO) = 5] and the comparable ROI (Fig. 5*A*) in WT mice [*n* (EE-WT) = 4, *n* (SE-WT) = 4] was undertaken (see Materials and Methods; Fig. 6*A* and 6*B*). This enabled us to quantify how expression of the marker proteins Iba-1 and CD68 varied in the ROI compared with the rest of the dLGN. Given the observations noted above, two specific properties were assessed for each protein: particle size and % area.

For the fold change analysis of “CD68 average particle size,” omnibus testing (univariate ANOVA with genotype and housing as fixed factors) revealed a significant genotype × housing interaction (*F*_(1,14)_ = 8.222, *p *=* *0.012). Pairwise comparisons indicated that fold change values for “CD68 average particle size” were significantly greater (*p *=* *0.001) in EE-KOs (1.513 ± 0.175, *n* =* *5) than SE-KOs (0.740 ± 0.302, *n* =* *5), but not significantly different (*p *=* *0.868) between EE-WTs (0.980 ± 0.030, *n* =* *4) and SE-WTs (0.969 ± 0.071, *n *=* *4; Fig. 6*C*). Fold change values were also significantly greater (*p *=* *0.021) in EE-KOs than EE-WTs, but not significantly different (*p *=* *0.168) between SE-KOs and SE-WTs (Fig. 6*C*).

For the fold change analysis of “CD68% area,” omnibus testing (univariate ANOVA with genotype and housing as fixed factors) revealed a significant genotype × housing interaction (*F*_(1,14)_ = 13.323, *p *=* *0.003). Pairwise comparisons indicated that fold change values for “CD68% area” were significantly greater (*p *<* *0.001) in EE-KOs (1.886 ± 0.169, *n *=* *5) than SE-KOs (0.565 ± 0.557, *n *=* *5), but not significantly different (*p *=* *0.748) between EE-WTs (1.106 ± 0.116, *n *=* *4) and SE-WTs (1.204 ± 0.136, *n *=* *4 Fig. 6*D*). Fold change values were also significantly greater (*p *= 0.013) in EE-KOs than EE-WTs, and significantly smaller (*p *=* *0.036) in SE-KOs than SE-WTs (Fig. 6*D*).

For the fold change analysis of “Iba-1 average particle size,” omnibus testing (univariate ANOVA with genotype and housing as fixed factors) revealed a significant genotype × housing interaction (*F*_(1,14)_ = 7.685, *p *=* *0.015). Pairwise comparisons indicated that fold change values for “Iba-1 average particle size” were significantly greater (*p *= 0.001) in EE-KOs (1.954 ± 0.323, *n *=* *5) than SE-KOs (0.986 ± 0.153, *n *=* *5), but not significantly different (*p *=* *0.954) between EE-WTs (0.980 ± 0.030, *n *=* *4) and SE-WTs (1.023 ± 0.056, *n *=* *4; Fig. 6*E*). Fold change values were also significantly greater (*p *=* *0.002) in EE-KOs than EE-WTs, but not significantly different (*p *=* *0.883) between SE-KOs and SE-WTs (Fig. 6*E*).

For the fold change analysis of “Iba-1% area,” omnibus testing (univariate ANOVA with genotype and housing as fixed factors) revealed a significant genotype × housing interaction (*F*_(1,14)_ = 17.587, *p *=* *0.001). Pairwise comparisons indicated that fold change values for “Iba-1% area” were significantly greater (*p *<* *0.001) in EE-KOs (1.895 ± 0.112, *n *=* *5) than SE-KOs (0.998 ± 0.274, *n *=* *5), but not significantly different (*p *=* *0.771) between EE-WTs (1.254 ± 0.134, *n *=* *4) and SE-WTs (1.206 ± 0.033, *n *=* *4; Fig. 6*F*). Fold change values were also significantly greater (*p *= 0.001) in EE-KOs than EE-WTs, but not significantly different (*p *=* *0.167) between SE-KOs and SE-WTs (Fig. 6*F*).

As an additional control, a fold change analysis was also undertaken on a region of dLGN at the dorsomedial (DM) extremity of labeled ipsilateral retinal terminals, a locus in which qualitatively, we did not see any obvious change in expression of Iba-1 and CD68 in response to EE (Figs. 4, 5), nor any notable pruning of RGC terminals (Fig. 2).

Consistent with qualitative observations, no significant effects were found between groups [SE-WT (*n* = 4), EE-WT (*n* = 4), SE-KO (*n* = 5), EE-KO (*n* = 5)], and fold change ratios (ROI/dLGN) were “centered” around a value of 1 (see Table 1). Omnibus testing (univariate ANOVA with genotype and housing as fixed factors) showed no main or interactive effect for genotype and/or housing in fold change analyses of “CD68 average particle size” (genotype: *F*_(1,14)_ = 0.066, *p *=* *0.801; housing: *F*_(1,14)_ = 0.055, *p *=* *0.818; genotype × housing interaction: *F*_(1,14)_ = 0.003, *p *= 0.956), “CD68% area” (genotype: *F*_(1,14)_ = 0.472, *p *=* *0.503; housing: *F*_(1,14)_ = 0.962, *p *=* *0.343 genotype × housing interaction: *F*_(1,14)_ = 0.530, *p *=* *0.479), “Iba-1 average particle size” (genotype: *F*_(1,14)_ = 0.012, *p *=* *0.909; housing: *F*_(1,14)_ = 0.261, *p *=* *0.618; genotype × housing interaction: *F*_(1,14)_ = 0.009, *p *= 0.928), or “Iba-1% area” (genotype: *F*_(1,14)_ = 3.109, *p *=* *0.100; housing: *F*_(1,14)_ = 0.385, *p *=* *0.545; genotype × housing interaction: *F*_(1,14)_ = 0.074, *p *=* *0.790).

**Table 1:** Fold change ratios of microglial CD68 and Iba-1 expression in the control region (dorsomedial border of ipsilateral retinal terminals) of Ten-m3 knock-out and wild-type mice dLGNs show no changes and are all centered around a value of 1

		**Fold change ratio (DM ROI**/**dlGN)**			
		Iba-1 “avg size”	Iba-1 “% area”	CD68 “avg size”	CD68 “% area”
**SE-WT**	Mean	0.98	1.15	0.98	1.07
	SD	0.14	0.14	0.17	0.15
**EE-WT**	Mean	0.94	1.19	0.94	1.03
	SD	0.09	0.25	0.09	0.27
**SE-KO**	Mean	0.99	0.94	1.00	1.29
	SD	0.12	0.20	0.39	0.49
**EE-KO**	Mean	0.96	1.03	0.98	1.02
	SD	0.08	0.28	0.22	0.35

Low-power images of Iba-1 and CD68 expression in the dLGNs of 3.5-week-old wild-type (WT, 4 animals per housing condition) and Ten-m3 knock-out (KO, 5 animals per housing condition) mice underwent image binarization as depicted in Figure 6, and values for average size and % area extracted for the whole dLGN and specific dorsomedial (D-M) ROI (dorsomedial border of ipsilateral retinal terminals in the dLGN). Fold change ratios (ROI/dlGN) were determined for each section. Presented are group means and associated standard deviation (SD). Dorsomedial (DM); standard enrichment (SE); environmental enrichment (EE).

Together, these results suggest that Iba-1 and CD68 expression are indeed different in EE-KOs compared with both SE-KOs and WTs, and that these changes are localized to the ventrolateral ROI. To better understand what underlies the observed EE-driven differences in expression of Iba-1 and CD68 at the ventrolateral border of retinal projections in Ten-m3 KO dLGNs, a higher resolution analysis which included quantification of individual morphologic features for all microglia in the KO and WT ROIs was performed (see Materials and Methods).

**Figure 7.**
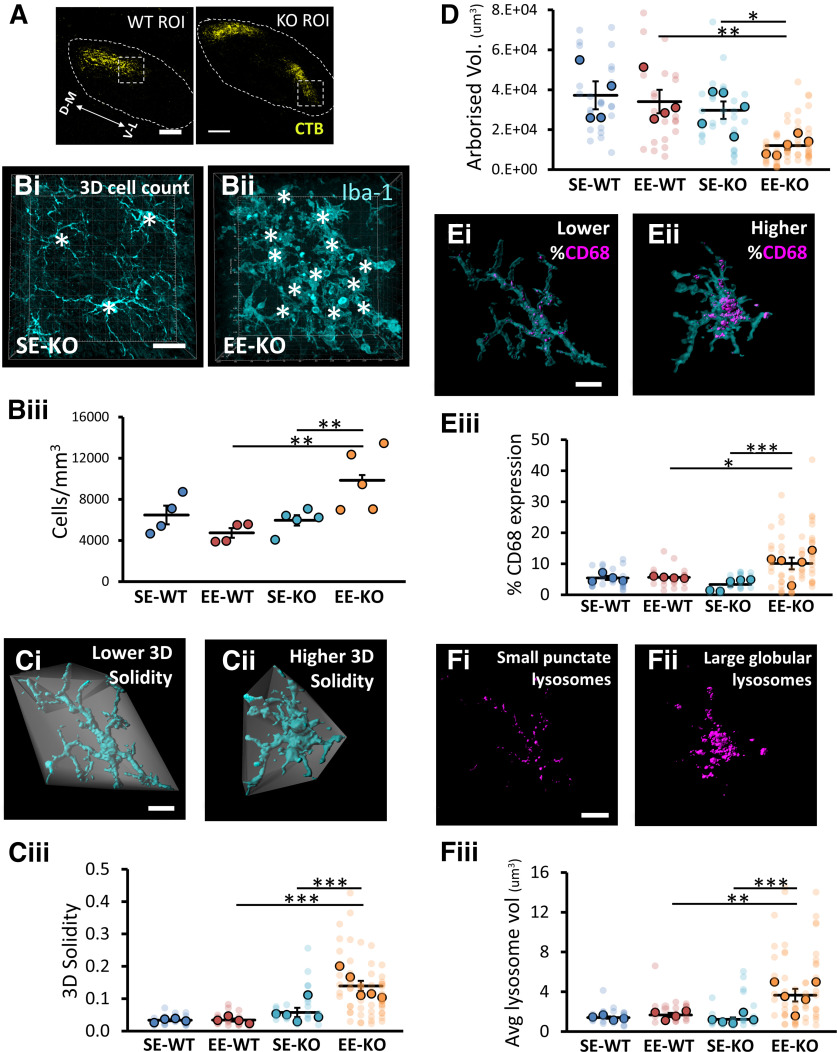
EE drives an “activated” profile in microglia located at the ventrolateral border of ipsilateral retinal terminals in the dLGN of Ten-m3 knock-out but not wild-type mice. ***A***, Region of interest (ROI) in WT and KO mice (boxed region; 116 × 116 μm), the ventrolateral border of ipsilateral retinal terminals (yellow) in the dLGN (outlined). ***B–F***, Depiction of the 3D analysis and resultant data from confocal z-stacks acquired of the ROI in 3.5-week-old SE-WTs (*n* = 4), EE-WTs (*n* = 4), SE-KOs (*n* = 5), and EE-KOs (*n* = 5). Microglia and their associated lysosomes were visualized using Iba-1 (cyan) and CD68 (magenta), respectively. The ROI was located via anterograde tracing of RGC axons in each mouse with Alexa Fluor-conjugated CTB, as shown in ***A***. ***Bi***, ***Bii***, Manual 3D cell count in a representative SE-KO (***B***) and EE-KO (***Bii***). Individual microglia are identified by super-imposed white asterixis. ***Biii***, Microglial densities were significantly greater in EE-KOs than SE-KOs (*p *=* *0.007) but were not significantly different between EE-WTs and SE-WTs (*p *=* *0.227). Microglial densities were also significantly greater in EE-KOs than EE-WTs (*p *=* *0.007), but not significantly different between SE-KOs and SE-WTs (*p *=* *0.699). ***Ci***, ***Cii***, Depiction of 3D solidity measure, more ameboid microglia (***Cii***; typically, more “activated”) possess a higher 3D solidity [(microglial volume)/(associated convex hull volume)] than more ramified microglia (***Ci***; typically, more “resting”). ***Ciii***, 3D solidity was significantly higher in EE-KOs compared with SE-KOs (*p *<* *0.001), but not significantly different between EE-WTs and SE-WTs (*p *=* *0.905). 3D solidity of microglia was also significantly greater in EE-KOs than EE-WTs (*p *<* *0.001), but not significantly different between SE-KOs and SE-WTs (*p *=* *0.301). ***D***, Arborized volume (convex hull volume, example shown in gray in ***Ci***, ***Cii***), is a proxy measure of the expanse of microglial processes. Arborized volume was significantly smaller in EE-KOs compared with SE-KOs (*p *=* *0.016), but not significantly different between EE-WTs and SE-WTs (*p *=* *0.669). Arborized volume of ROI microglia was also significantly smaller in EE-KOs than EE-WTs (*p *=* *0.006), but not significantly different between SE-KOs and SE-WTs (*p *=* *0.294). ***Ei***, ***Eii***, Depiction of %CD68 expression measure ([CD68 volume/Iba-1 volume] × 100%), a contrast is given between a lower %CD68 microglia (***Ei***) versus a higher %CD68 microglia (***Eii***). ***Eiii***, %CD68 expression was significantly greater in EE-KOs compared with SE-KOs (*p *=* *0.001), but not significantly different between EE-WTs and SE-WTs (*p *=* *0.925). %CD68 expression was also significantly greater in EE-KOs than EE-WTs (*p *=* *0.021), but not significantly different between SE-KOs and SE-WTs (*p *=* *0.237). ***Fi***, ***Fii***, Depiction of average lysosome volume measure (rendered CD68 lysosomes shown only) – a contrast is given between a lower average lysosome volume microglia with small punctate lysosomes (***Fi***) and a larger average lysosome volume microglia with large globular lysosomes (***Fii***). ***Fiii***, Average lysosome volume was significantly greater in EE-KOs compared with SE-KOs (*p *<* *0.001), but not significantly different between EE-WTs and SE-WTs (*p *=* *0.656). The average lysosome volume was also significantly greater in EE-KOs than EE-WTs (*p *=* *0.003), but not significantly different between SE-KOs and SE-WTs (*p *=* *0.757). Results shown are of pairwise comparisons. Intensely colored circles represent data values from independent samples: an aggregate value (see Materials and Methods) if individual cell measures were taken. Faint data points, grouped in columns according to sample identity, represent data values from single-cell measurements. Four animals per housing condition for WTs and five animals per housing condition for KOs. Mean bars are depicted. Error bars show SEM. Ventrolateral (V–L); dorsomedial (D–M). Scale bars: 100 μm (***A***), 20 μm (***B***), 10 μm (***C–F***). *p ≤* *0.05, **p ≤* *0.01, ***p ≤* *0.001.

Cell densities of microglia in WT and KO ROIs (Fig. 7*A*) were first quantitatively assessed using a three-dimensional (3D) manual count (see Materials and Methods; Fig. 7*Bi*,*Bii*). Omnibus testing (univariate ANOVA with genotype and housing as fixed factors) revealed a significant genotype × housing interaction (*F*_(1,14)_ = 9.294, *p *=* *0.009). Pairwise comparisons indicated that microglial densities were indeed significantly greater (*p *=* *0.007) in EE-KOs (9855 ± 1334 microglia/mm^3^, *n *=* *5) than SE-KOs (5960 ± 505 microglia/mm^3^, *n *=* *5), but not significantly different (*p *=* *0.227) between EE-WTs (4733 ± 466 microglia/mm^3^, *n *=* *4) and SE-WTs (6475 ± 909 microglia/mm^3^, *n *=* *4; Fig. 7*Biii*). Microglial densities were also significantly greater (*p *=* *0.007) in EE-KOs than EE-WTs, but not significantly different (*p *=* *0.699) between SE-KOs and SE-WTs (Fig. 7*Biii*).

The morphologies of microglia in WT and KO ROIs (Fig. 7*A*) were quantitatively assessed through the measures of “3D solidity” and arborized volume (see Materials and Methods; Fig. 7*Ci*,*Cii*). 3D solidity facilitated a quantitative distinction between more ramified (lower 3D solidity; Fig. 7*Ci*) versus more amoeboid (higher 3D solidity; Fig. 7*Cii*) morphologies. Omnibus testing for this measure (univariate ANOVA with genotype and housing as fixed factors) revealed a significant genotype × housing interaction (*F*_(1,14)_ = 9.785, *p *=* *0.007). Pairwise comparisons indicated that 3D solidity was significantly greater (*p *<* *0.001) in EE-KOs (0.139 ± 0.016, *n *=* *5) when compared with SE-KOs (0.058 ± 0.014, *n *=* *5), but not significantly different (*p *=* *0.905) between EE-WTs (0.034 ± 0.005, *n *=* *4) and SE-WTs (0.033 ± 0.003, *n *=* *4; Fig. 7*Ciii*). 3D solidity was also significantly greater (*p *<* *0.001) in EE-KOs than EE-WTs, but not significantly different (*p *=* *0.301) between SE-KOs and SE-WTs (Fig. 7*Ciii*).

Arborized volume (convex hull volume, see Materials and Methods; convex hull presented in Fig. 7*Ci*,*Cii*, gray) was used as a proxy measure for the expanse of microglial processes: microglia have been shown to retract their processes when activated (Schafer et al., 2012). For this measure, omnibus testing (univariate ANOVA with genotype and housing as fixed factors) revealed no significant genotype × housing interactions (*F*_(1,14)_ = 2.244, *p *=* *0.156) but did reveal significant effects of genotype (*F*_(1,14)_ = 9.231, *p *=* *0.009) and housing (*F*_(1,14)_ = 4.618, *p *=* *0.050). Pairwise comparisons indicated that arborized volume was significantly smaller (*p *=* *0.016) in EE-KOs (11 957 ± 2067 μm^3^, *n *=* *5) compared with SE-KOs (29 721 ± 4401 μm^3^, *n *=* *5), but not significantly different (*p *=* *0.669) between EE-WTs (34 053 ± 5873 μm^3^, *n *=* *4) and SE-WTs (37 224 ± 7009 μm^3^, *n *=* *4; Fig. 7*D*). Arborized volume of ROI microglia was also significantly smaller (*p *=* *0.006) in EE-KOs than EE-WTs, but not significantly different (*p *=* *0.294) between SE-KOs and SE-WTs (Fig. 7*D*).

The CD68 expression of microglia in the WT and KO ROIs (Fig. 7*A*) was quantitatively assessed through the measures of %CD68 expression and average lysosome volume. %CD68 was calculated as the percentage of individual microglial volume (Iba-1 labeling) taken up by CD68 expression (Fig. 7*Ei*,*Eii*). Omnibus testing for this measure (univariate ANOVA with genotype and housing as fixed factors) revealed a significant genotype × housing interaction (*F*_(1,14)_ = 7.339, *p *=* *0.017). Pairwise comparisons indicated that %CD68 expression was significantly greater (*p *=* *0.001) in EE-KOs (10.1 ± 1.9%, *n *=* *5) than SE-KOs (3.3 ± 0.8%, *n *=* *5), but not significantly different (*p *=* *0.925) between EE-WTs (5.6 ± 0.2%, *n *=* *4) and SE-WTs (5.4 ± 0.6%, *n *=* *4; Fig. 7*Eiii*). Percentage CD68 expression was also significantly greater (*p *=* *0.021) in EE-KOs than EE-WTs, but not significantly different (*p *=* *0.237) between SE-KOs and SE-WTs (Fig. 7*Eiii*).

Average lysosome volume (for details, see Materials and Methods) was included to build on the findings with the %CD68 measure, driven by the qualitative observations that the size of the CD68 clumps (“lysosomes”) was generally larger in EE-KOs (Figs. 4*Dii* or 7*Fii* for single microglia CD68 expression) than SE-KOs (Figs. 4*Cii* or 7*Fi* for single microglia CD68 expression). Omnibus testing (univariate ANOVA with genotype and housing as fixed factors) revealed a significant genotype × housing interaction (*F*_(1,14)_ = 7.358, *p *=* *0.015). Pairwise comparisons indicated that the average lysosome volume of individual ROI microglia was significantly greater (*p *<* *0.001) in EE-KOs (3.67 ± 0.63 μm^3^, *n *=* *5) compared with SE-KOs (1.23 ± 0.19 μm^3^, *n *=* *5), but not significantly different (*p *=* *0.656) between EE-WTs (1.67 ± 0.22 μm^3^, *n *=* *4) and SE-WTs (1.40 ± 0.11 μm^3^, *n *=* *4; Fig. 7*Fiii*). The average lysosome volume of individual ROI microglia was also significantly greater (*p *=* *0.003) in EE-KOs than EE-WTs, but not significantly different (*p *=* *0.757) between SE-KOs and SE-WTs (Fig. 7*Fiii*).

An additional statistical analysis was done of microglial volumes (Iba-1 labeling) in WT and KO ROIs (Fig. 7*A*) to determine whether the results found with the %CD68 measure or 3D solidity (both utilizing microglial volume as a component of the measure, see Materials and Methods) could be attributed to changes in overall microglial volume in the different cohorts. This was found to be an unlikely explanation as omnibus testing (univariate ANOVA with genotype and housing as fixed factors) revealed no significant genotype × housing interactions (*F*_(1,14)_ = 0.094, *p *=* *0.763) and no significant effects of genotype (*F*_(1,14)_ = 2.220, *p *=* *0.158) or housing (*F*_(1,14)_ = 1.637, *p *=* *0.222).

Collectively we have shown that that EE drives an “activated” profile in microglia located at the ventrolateral border of ipsilateral RGC terminals in Ten-m3 KO mice, the locus of greatest ectopic RGC terminal pruning. An equivalent response to EE was not observed in age-matched WT controls, consistent with the anatomical and behavioral findings of this study. This phenotype is present at 3.5 weeks of age, demonstrating a temporal correlation between the correction of the anatomical miswiring and the rescue of visually mediated behavior in KO mice.

## Discussion

This study has revealed that with EE commenced from a few days before birth until just 3.5 weeks postnatal is sufficient to drive improvements to visually-mediated behavior in a model of neural miswiring where visual function is usually profoundly impaired for life. This behavioral rescue was associated with significant degree of corrective pruning of the miswired inputs at their most visuotopically inappropriate locus. Notably, EE until 3.5 weeks postnatal also drove increased microglial densities at this same locus and induced microglia (at this locus) to take on what is typically considered an “activated” profile. No effects of EE on microglia were found in age matched WT controls. These results highlight microglia recruitment as a candidate cellular mechanism through which EE from birth drives corrective pruning and functional rescue of miswired neural circuits.

Our study shows that ∼3.5-week-old WT mice respond reliably and rapidly to the looming stimulus. Although this response has been well characterized for adult mice (Yilmaz and Meister, 2013; Zhao et al., 2014; Blok et al., 2020), its development has only recently been described. Other groups report that this response is not reliable before P20 but becomes robust between three and four weeks of age (Narushima et al., 2016; Chen et al., 2022). Our data agree with these observations. Notably, mice at this age have only had their eyes open and been fully mobile for around two weeks. This age slightly precedes the peak of the cortical critical period and maturation of visual cortical circuitry (Gordon and Stryker, 1996; Huang et al., 1999). Our work suggests that the circuitry allowing subsecond responses to this stimulus is established by the age tested. Moreover, no significant changes were observed in EE- versus SE-WTs. This may indicate that there is a ceiling effect by this stage. Although both visual cortical (driven primarily by retinogeniculate input) and retinocollicular streams have been shown to contribute to this characteristic flight behavior in adults (Zhao et al., 2014; Wei et al., 2015), to what degree these pathways drive the response at this early age is not yet known (see also Blok et al., 2020 for further discussion).

In contrast, SE-KO mice did not respond rapidly or reliably to the stimulus. The simplest interpretation of this is that they were unaware of the stimulus which can be perceived only visually. Interestingly, ∼3.5 weeks of EE from birth was sufficient to induce a significant improvement in the reliability of the response. Although slower than WT responses, there was a marked rescue in terms of processing time and flight trajectories. This is consistent with what was observed following six weeks of EE from birth (Blok et al., 2020). While again it is not possible to demonstrate that this was due exclusively to an improvement in vision, the fact that this was accompanied by the pruning of miswired ipsilateral retinogeniculate inputs that have been previously shown to cause profound visual deficits (Leamey et al., 2007; Merlin et al., 2013), strongly suggests that there may be a relationship. Alternatively, since Ten-m3 is expressed in a variety of brain areas (for review, see Leamey and Sawatari, 2019), there may be other phenotypes in SE-KO mice that are also improved by exposure to EE (see Blok et al., 2020 for further discussion). The behavioral rescue in EE-KOs at 3.5 weeks cannot be attributed merely to an acceleration of visual circuitry development, as SE-KOs at six weeks and older were previously found to be unresponsive to the stimulus (Blok et al., 2020). Since the looming escape response is only just becoming reliable in WTs at three to four weeks of age (Narushima et al., 2016; Chen et al., 2022) its absence in SE-KOs at the stage examined suggests that it is defective from the outset in this group.

A primary finding was that the corrective pruning of miswired projections in EE-KOs was underway at ∼3.5 weeks of age. This is broadly consistent with our previous work showing that there is an early critical period when EE can induce corrections in miswired retinogeniculate projections (Eggins et al., 2019). Curiously, the effect resulting from six weeks of EE was observed if enrichment was commenced just before birth, but not at weaning (P21). At one level this is surprising, given that here we show the corrective pruning process is underway at 3.5 weeks (4 d after weaning, P25). It should be noted, however, that a substantial amount of elimination of miswired projections has already occurred by this time point: it is possible that EE may have to be in place well before the corrective pruning commencing, to facilitate any significant enhancements (i.e., upstream molecular processes may have their own “critical periods” of engagement). Further, comparison with the Ten-m3 KOs that were exposed to six weeks of EE from birth revealed that significant pruning occurs beyond the 3.5-week time point examined here. Interestingly, however, despite continued exposure to EE for six weeks, the pruning was only partial. Together, these scenarios point to the notion that there is likely a narrow developmental window during which EE can trigger corrective pruning and drive recovery of function. Further studies which investigate these possibilities in more detail are warranted.

Another major finding of this study was that early exposure EE for 3.5 weeks drives an “activated” profile in microglia located at the region of greatest ectopic RGC terminal removal. In 3.5-week-old EE-KOs, an increase in microglial density was apparent around the most ectopically mistargeted projections (which were also those targeted in the EE-driven corrective pruning; see also Eggins et al., 2019). Upregulation of CD68 and Iba-1 expression, phenotypes previously found to be heightened in microglia actively undergoing developmental pruning of RGC terminals in an earlier developmental stage (P5–P9; Schafer et al., 2012), were also apparent at this same locus. These results strongly suggest microglia as a candidate mechanism through which EE drives the removal of miswired visual circuitry in Ten-m3 KO mice. Central to microglia’s role as mediators of neuroplasticity, is their capacity to engulf (e.g., via phagocytosis) and remove neuronal elements (Weinhard et al., 2018; Vukojicic et al., 2019). It is interesting to consider whether this, and/or an alternate phagocytosis independent mechanism (Cheadle et al., 2020) may be involved.

The focal microglial activation characterized by this study appears to be a distinct process from the normal cellular processes of developmental refinement. In WT mice, heightened RGC terminal pruning by microglia occurs in two major developmental waves: an early wave at ∼P5–P9 (Schafer et al., 2012) and a late wave at ∼P40–P50 (Schafer et al., 2016). The time point of the EE-driven microglial activation characterized here (P25), however, coincides with neither. The WT retinogeniculate synapse has been shown to enter a period of vision-dependent refinement between P20 and P30 (Hooks and Chen, 2006, 2008; Cheadle et al., 2020), a time point which does overlap with our assessment period. Retinogeniculate axons during this period, however, have only been reported to undergo small-scale re-adjustments to their bouton positioning in response to dark rearing (Hong et al., 2014). Large scale structural changes to the axonal scaffold, such as those likely needed to mediate the corrective pruning effect observed by this study, are not seen till much later (Hong et al., 2014). Further, while microglia have been implicated in the vision-sensitive refinement period, they are not reported to undergo significantly heightened levels of RGC terminal pruning during this time (Cheadle et al., 2020). This is consistent with our observation that microglial activation was not visually apparent in 3.5-week-old SE-WTs or EE-WTs. It should be pointed out, however, that EE may activate microglia in WTs at different developmental stages, but assessment of this was beyond the scope of our study.

Previous work has demonstrated an effect of EE on microglia, alterations in microglial-mediated phagocytosis, cell density, morphology, CD68 expression, and cytokine-inflammatory profile (for review, see Augusto-Oliveira and Verkhratsky, 2021). In many of these studies, however, EE induced changes in microglia were characterized following a specific immune insult (Garofalo et al., 2017; Cope et al., 2019; GF Gomes et al., 2019) or inflammation associated with neurodegenerative disease (Xu et al., 2016; Hase et al., 2017; Wassouf et al., 2018; Ziegler‐Waldkirch et al., 2018). Regionally specific changes to microglial morphology and densities have been described (Ehninger and Kempermann, 2003; Ziv et al., 2006; Ali et al., 2019; Singhal et al., 2021), although in a somewhat limited manner. To the best of our knowledge, our study is the first to reveal the EE dependent recruitment of activated microglia, seemingly to promote repair of profoundly miswired neural circuits.

It remains possible in our model that EE could compensate for potential deficiencies in microglial function in mice missing Ten-m3. As noted previously, heightened pruning of retinogeniculate projections by microglia occurs at around P5–P9 as part of the normal developmental segregation of ipsilateral and contralateral eye inputs within the dLGN (Schafer et al., 2012). Although we did not investigate this age-point here, previous work has shown that this process remains intact, and occurs over the same developmental timeline in Ten-m3 KO mice (Glendining et al., 2017). This suggests that microglia, at least in their RGC terminal pruning capacities, are unlikely to be directly affected by the lack of Ten-m3.

Results from this study suggest that EE can facilitate the identification and/or removal of miswired visual circuits in the Ten-m3 KO mouse. Which aspects of these processes are particularly affected by EE, however, is yet to be determined. Conceivably, a richer visual experience could facilitate the differentiation of appropriately wired contralateral inputs from miswired ipsilateral inputs via activity-dependent processes. Cortical feedback loops to the dLGN, which are maturing around this time (Jurgens et al., 2012; Seabrook et al., 2013) and have the capacity to functionally influence retinogeniculate connectivity (Thompson et al., 2016), might also be involved.

Alternatively, or in concert, heightened activity because of EE may affect upstream processes to increase or otherwise “prime” microglial activation. The molecular cues that enable interaction between neurons and microglia during normal development remain under investigation but have been shown to involve neural activation (Tremblay et al., 2010; Schafer et al., 2012) as well as members of the complement family (Stevens et al., 2007; Vukojicic et al., 2019; C Wang et al., 2020), purinergic signaling mechanisms (Sipe et al., 2016), and TREM-2 (Filipello et al., 2018). EE has also been shown to upregulate neurotrophic factors such as BDNF (Pham et al., 2002; Cancedda, 2004) as well as IGF1 (Ciucci et al., 2007) in the developing visual pathway and they may also play a role here. Interestingly, both BDNF and IGF1 can be produced by microglia (C Gomes et al., 2013; Suh et al., 2013; Myhre et al., 2019) and can induce phenotypic changes in these cells (Zhang et al., 2014). For example, while IGF1 has been most widely associated with promoting conversion to a resting state in these cells (for review, see Labandeira-Garcia et al., 2017), there is evidence that it can also induce activation (Falomir-Lockhart et al., 2019), suggesting the trophic factor’s effect on microglia could change under different circumstances such as age, pathologic condition, brain area, or experience. It will be of interest to determine to what extent EE activates microglia via these processes and/or factors, or through other distinct mechanisms.

It should be noted that while we generally describe the intervention as EE from birth, for animal welfare and logistical reasons, it was necessary to commence the EE period from a few days before birth to prevent stress during the immediate postnatal period. Further, describing it in this way is useful as it clearly contrasts it with other studies where EE is commenced significantly after birth. It should be acknowledged, however, that it is possible that EE in the last few prenatal days could have an effect on the developing brain. The impact on pruning of the circuit being studied here is likely to be minimal, as retinal axons are just growing in to the dLGN at this time (Godement et al., 1984), and we previously showed that EE from just before birth until P7 had no impact on the targeting or refinement of ipsilateral retinogeniculate projections in Ten-m3 KOs (Eggins et al., 2019). Nevertheless, the potential for prenatal EE to impact brain development is important and should be kept in mind when interpreting these results.

Finally, evidence exists for the etiological involvement of axonal guidance and pathfinding errors in neurodevelopmental disorders such as ASD (McFadden and Minshew, 2013). Given this, the identification of a potential cellular mechanism which can drive corrective pruning as well as improve function, and that can be activated by experience during a window of early postnatal life, may have important implications for the development of therapies targeting neurodevelopmental disorders. Further, beneficial changes following interventions which modulate experience during early development have already been reported for children diagnosed with, or at risk of, ASD (Woo and Leon, 2013; Woo et al., 2015; Aronoff et al., 2016; Whitehouse et al., 2021) with generally greater impact observed when commenced earlier in development (McDonald et al., 2018). Our results provide a potential additional avenue and a clear cellular/mechanistic-level justification to develop or augment such therapies as well as to determine key timepoints for their implementation.

In conclusion, we show that 3.5 weeks of EE, commencing several days before birth, is sufficient to drive corrective pruning of neural projections that were profoundly miswired because of genetic deletion. Further, we have shown that microglial density and activation profile is increased at the primary locus of axon elimination, highlighting microglia as likely having mechanistic involvement in mediating this effect. The corrective pruning was also associated with the recovery of an ethologically appropriate visually-mediated behavior. To the best of our knowledge, this study provides the first results suggesting that EE can activate microglia to drive removal of miswired circuitry. Further, this EE-induced corrective pruning process was highly selective both spatially and temporally and was not activated in the absence of miswired inputs. These findings provide a potential avenue for the development of novel therapies to treat neurodevelopmental disorders involving early neural miswiring (for review, see McFadden and Minshew, 2013), such as ASD. Our work adds to a growing awareness of the critical links between experience and microglial activation, brain health, development and dysfunction.

## Synthesis

Reviewing Editor: Isabel Perez-Otaño, Instituto de Neurociencias Decisions are customarily a result of the Reviewing Editor and the peer reviewers coming together and discussing their recommendations until a consensus is reached. When revisions are invited, a fact-based synthesis statement explaining their decision and outlining what is needed to prepare a revision will be listed below. The following reviewer(s) agreed to reveal their identity: NONE. Note: If this manuscript was transferred from JNeurosci and a decision was made to accept the manuscript without peer review, a brief statement to this effect will instead be what is listed below.

This study work builds on previous work by the authors and provides further evidence implicating microglia-mediated mechanisms in axonal pruning to correct mis-wiring. The revised manuscript addresses most issues raised by reviewers, with only minor concerns that we hope can be now taken into consideration (see below)

REVIEWER COMMENTS

The authors made a huge effort to address the reviewers comments, doubts and criticisms, particularly those related to statistics. As a result, the article has greatly improved. However, there are still some issues that need to be corrected before publication:

1. The authors state several times along the manuscript that EE leads to “correction or repair of miswired circuits”. Writing the word “repair” may be lead to the idea that connections that were not established because the axons projected in wrong areas, are occurring in the EE-mutant but this is not what they demonstrate. What they really demonstrate is the existence of partial “pruning” of miswired axons. Therefore, this should be amended all along the manuscript by replacing the words “correction” and “repair” for “elimination/pruning (or similar) of axon terminals”.

2. The images shown in Fig4A, Fig6A and Fig7A are all the same picture. Examples from different animals should be shown in each Figure.

3. They should try to provide a hypothesis of why do they think that the microglia-mediated pruning is partial, why microglia does not eliminate all the misprojected terminals? Why only some of them?

## Author Response

REVIEWER COMMENTS

The authors made a huge effort to address the reviewers comments, doubts and criticisms, particularly those related to statistics. As a result, the article has greatly improved. However, there are still some issues that need to be corrected before publication:

1. The authors state several times along the manuscript that EE leads to “correction or repair of miswired circuits”. Writing the word “repair” may be lead to the idea that connections that were not established because the axons projected in wrong areas, are occurring in the EE-mutant but this is not what they demonstrate. What they really demonstrate is the existence of partial “pruning” of miswired axons. Therefore, this should be amended all along the manuscript by replacing the words “correction” and “repair” for “elimination/pruning (or similar) of axon terminals”.

**These terms have been replaced through the text as requested.

2. The images shown in Fig4A, Fig6A and Fig7A are all the same picture. Examples from different animals should be shown in each Figure.

**This was done as the images were mostly included for orientation, but have now been replaced with alternative examples as requested

3. They should try to provide a hypothesis of why do they think that the microglia-mediated pruning is partial, why microglia does not eliminate all the misprojected terminals? Why only some of them?

**While we agree that this is an interesting point, we did not dwell on it this paper which takes a snapshot of a time when the pruning is in process to determine the mechanisms. It is an important topic for future work and we have added a few lines to the Discussion to point this out (pp31-32).
